# Development of a diagnostic assay by three-tube multiplex real-time PCR for simultaneous detection of nine microorganisms causing acute respiratory infections

**DOI:** 10.1038/s41598-022-15543-6

**Published:** 2022-08-03

**Authors:** Xi-Wen Jiang, Tao-Sheng Huang, Long Xie, Si-Ze Chen, Shi-Dong Wang, Zhi-Wen Huang, Xin-Yu Li, Wei-Ping Ling

**Affiliations:** 1 Research Institute, DAAN Gene Co., Ltd., No. 19 Xiangshan Road, Guangzhou, China; 2 The Medicine and Biological Engineering Technology Research Center of the Ministry of Health, Guangzhou, China; 3 Clinical and Translational Research Institute, Faculty of Medical Sciences, Newcastle University, Newcastle upon Tyne, UK; 4 Central Laboratory, The First Affiliated Hospital of Guangdong Pharmaceutical University, Guangzhou, China; 5 The Precise Therapy Engineering Technology Research Center of Guangdong Province for Esophageal Cancer, Guangzhou, China

**Keywords:** Biological techniques, Microbiology, Molecular biology

## Abstract

Acute respiratory infections are widespread in vulnerable populations of all ages and are characterized by a variety of symptoms. The underlying infection can be caused by a multitude of microorganisms, including viruses and bacteria. Early detection of respiratory infections through rapid pathogen screening is vital in averting infectious respiratory disease epidemics. This study utilized a multiplex real-time PCR system to develop a three-tube reverse transcription-PCR (RT-PCR) assay, enabling simultaneously detect nine respiratory pathogens, including: influenza A and B, adenovirus, respiratory syncytial virus (RSV), *Streptococcus pneumoniae*, *Legionella pneumophila*, *Haemophilus influenzae*, *Chlamydia pneumoniae*, and *Mycoplasma pneumoniae*. This technique utilizes a one-step assay, with specifically designed TaqMan primer–probe sets combined in the same tube. This assay provided rapid and simplified detection of the nine prevalent pathogens, as well as increased sensitivity and reduced cross-contamination. This assay was evaluated using 25 related viral/bacterial strains as positive references, the other 25 irrelevant strains as negative controls, and clinical specimens from 179 patients. All positive strains were detected with no amplification of the non-target microorganism mixtures and the assay’s detection limits ranged between 250–500 copies/ml (1.25–2.5 copies/reaction). A total of 167 (93.3%) samples tested positive for at least one of the pathogens identified; 109 of these samples were from patients confirmed to have RSV infections. The diagnostic accuracy of our assay was further confirmed by matching results from classical direct immunofluorescence assay and nucleotide sequencing. These data demonstrate the innovative multiplex real-time PCR assay as a promising alternative to the current approaches used for early screening of acute respiratory infections.

## Introduction

Acute respiratory infections present one of the most serious threats to global public health and are established to be a significant cause of morbidity and mortality in children and immunocompromised adults^1,2^. Epidemiological data suggested lower respiratory tract infections were responsible for 2.7 million deaths in 2015, ranking it third in terms of disease burden^3^. Compared to other age groups, children under 5 years of age accounting for 12–19% of all deaths in patients with lower respiratory tract infections; pneumococcal pneumonia has been identified as being the causative agent behind 55.8% of this respiratory tract associated^3,4^. Therefore, an accurate and rapid diagnosis of the causative pathogens of infections is crucial to the selection of the appropriate therapeutics^5^; this will minimize the use of unnecessary antibiotics and ensure the swift implementation of the appropriate treatment^6^.

Acute respiratory infections are attributable to a variety of pathogens, including bacteria, viruses, and mycoplasmas. They are associated with a broad spectrum of symptoms, such as cough, fatigue, and fever^7^. Viral infection accounts for appropriate 80% of acute respiratory infections, with influenza virus^8^, respiratory syncytial virus (RSV)^9^, and respiratory adenovirus^6^ being the most common pathogens. Large outbreaks of viral respiratory tract infections lead to the infection of a greater population of people, resulting in infection in at-risk groups who are more likely to develop significant morbidities^3,4,10^. In addition to this, bacterial pneumonia has become a serious public health issue to the increased morbidity of the infection which can often lead to hospitalization and mortality in these populations^11^; highly infectious pathogenic bacteria, including *Streptococcus pneumonia*^12^, *Haemophilus influenza*^13^, *Mycoplasma pneumoniae*, *Chlamydia pneumoniae*, and *Legionella pneumophilia*^14^, have all been demonstrated to be causative agents. In short, acute respiratory infections represent a category of infectious disease caused by multiple pathogenic agents, this increases the difficulty of diagnosis and complicates treatment strategies due to the diversity and complexity of the infectious pathogens^15^. Thus, this highlights the importance of establishing a rapid, effective, and accurate screening approach for the identification of causative agents of acute respiratory infections^16^.

Bacterial/viral cultures and serological tests are the current gold standard for the diagnosis of acute respiratory infections^17^; they often lack sensitivity and are time and labour-consuming, leading to delays in treatment and the use of ineffective therapeutics^18^. The immunofluorescence assays, for example the direct fluorescent antibody (DFA) assay, enable rapid detection of respiratory virus antigens by utilizing a fluorescent-tagged antibody directly against target viruses, and are commonly deployed for pathogen screening in the clinical frontline^19^. The disadvantages of these methods, such as the lack of ultra-sensitivity caused by background staining and the rapid weakening of fluorescent signal, have led to the demand for comprehensive solutions for the detection of multiple pathogens-involved in clinical cases presenting as acute respiratory infections^20^. Polymerase chain reaction (PCR) has proven to be fast, low-cost, and sensitive method of utilizing nucleic acid for the detection of various microorganisms^21^. In 1992, Harris’s group developed a reverse transcription (RT)-based PCR method for the rapid, sensitive and specific detection of RSV in human samples^22^. Building on this, a multiplex RT-PCR system was developed, which enabled the simultaneous detection of nine different acute respiratory infection causing microorganisms in single tubes; these included bacteria and RNA and DNA based viruses^23^. A similar study was conducted with the development of a multiplex strategy for the simultaneous detection of 18 different respiratory viruses plus 3 bacteria^18^. Despite achieving high-throughput and conveniency, these Agarose gel electrophoresis-based conventional multiplex PCR methods required post-PCR analysis, leading to deficiencies in sensitivity and specificity^24^. With the demand of increased sensitivity, a quantitative TaqMan PCR method has been suggested for use in a one-tube nested, real-time PCR detection method, with numerous studies alluding to the potential of such a detection method^25^. Despite its many advantages, the dual sets of primers required for the multiplex fluorescent nested PCR method make it more difficult to design and optimise the experimental system, whilst also making it inconvenient to perform on large-scale clinical specimen screening. In addition, the application of multiplex real-time nested PCR for the detection of respiratory pathogens other than viruses is not yet commonly reported^18,25^. Regarding this, an innovation combining high sensitivity and specificity, low-cost, operational simplicity and practicability is required to modify multiplex TaqMan PCR in order to permit the rapid and precise testing of a wide spectrum of causative microorganisms in acute respiratory infections.

In the present study, a TaqMan probes-based one-step multiplex PCR assay was developed for the simultaneous detection of the leading causes of viral and bacterial acute respiratory infections; these included: influenza virus A (IVA), influenza virus B (IVB), adenovirus, RSV, *Streptococcus pneumoniae*, *Legionella pneumophila*, *Haemophilus influenzae, Chlamydia pneumoniae*, and *Mycoplasma pneumoniae.* The novelty of this laboratory-developed testing originated in the capacity to increase diagnostic positivity and identification of co-infections and reduce cross-interference by qualitatively detecting the cleverly pooled target pathogens from a wide range of microorganism types and subtypes and distinguishing them by utilizing specially designed TaqMan probes that have been individually labelled with different fluorescent dyes to target the gene sequences of the pathogens of interest. Every four probes, along with their corresponding primers, were combined in each reaction tube to match four colours of fluorescent channels, which is the regular configuration of most existing real-time PCR instruments, to achieve the simultaneous detection of up to three pathogens, one-third of the total nine above covered in this assay, therefore possessed practicality, simplicity and cost-effectiveness in clinical laboratories. This study aimed to utilize this novel multi-target, rapid and economic molecular tool to ameliorate the burden of frontline clinics diagnosis and epidemiological containment in acute respiratory infections.

## Results

### Establishment of standard curves of the multiplex real-time RT-PCR

**Figure 1:**
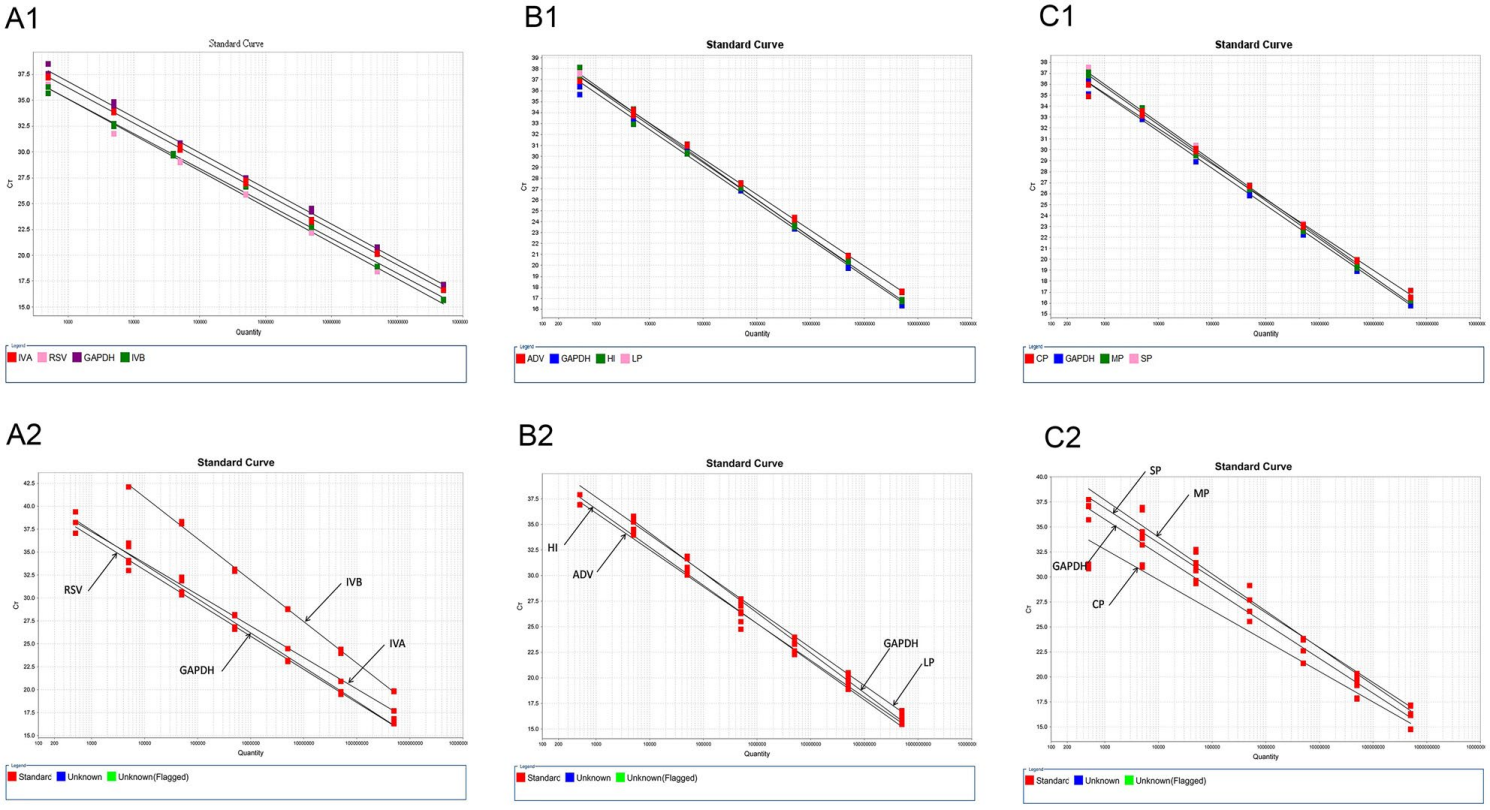
Standard curves of multiplex real-time PCR. A1, B1 and C1 are the standard curves conducted using single portions of each pathogenic nucleic acids, serial tenfold dilutions (5 × 10^8^ to 5 × 10^2^ copies/ml) by the four-plex real-time PCR assay in tubes A, B and C, respectively. A2, B2, and C2 are the standard curves generated under the condition of serial tenfold dilutions of the mixed nucleic acids (5 × 10^8^ to 5 × 10^2^ copies/ml) from all target pathogens by the multiplex real-time PCR assay in tubes A, B and C, respectively.

Prior to multiplexing of pathogens, the multiplex real-time PCR assay was initially optimised and validated using single template nucleic acids extracted from the previously listed 25 viral and bacterial strains carrying the target genes. Data from optimisation on PCR amplification revealed a MMLV reverse transcription concentration of 0.4 U/µl and an annealing temperature of 55 °C being the optimized conditions (Supplemental Fig. S1). The linearity of the multiplex assay was then assessed using serial tenfold dilutions of each pathogenic nucleic acid sample (from 5 × 10^8^ to 5 × 10^2^ copies/ml) in an individual format. After confirmatory data showed no cross-interference between multiple primers and probes, a mixture of nucleic acids from all nine target pathogens was amplified by the multiplex reaction to assess the orthogonality in multiplexing. Figure 1 shows the profiles of single-pathogen (A1, B1, C1) and multiple-pathogen (A2, B2, C2) standard curves. The assay correlated well for individual gene detection in all three tubes, with the correlation coefficient (R^2^) and amplification efficiency (E) values displayed in Table 1. However, when mixed pathogen nucleic acids were amplified in the multiplex PCR system, the efficiency of this type of PCR reaction was somewhat inhibited compared to the individual pathogen amplifications, as shown in Table 1 for R^2^ and E values that were affected to varying degrees but generally acceptable, suggesting that these poorer amplification efficiencies did not disrupt the ability of the multiplex PCR assay to detect unknown samples. Overall, data indicate that the individual pathogenic assay in the multiplex RT-PCR system was linear in the range of 5 × 10^8^–5 × 10^2^ template copies/ml, generating acceptable corresponding R^2^ and E values.

**Table 1:** The values of R^2^ and E for the target pathogens analysed in the three-tube multiplex real-time PCR.

Tube	Target	R^2^		E (%)	
		Single pathogen	Multi pathogen	Single pathogen	Multi pathogen
A	IVA	1	0.991	95.492	95.672
	IVB	0.997	0.997	97.453	68.07
	RSV	0.998	0.992	94.149	85.302
	GAPDH	0.998	0.993	95.412	89.174
B	Adenovirus	0.999	0.993	102.079	89.547
	*Haemophilus influenzae*	0.997	0.996	96.732	85.275
	*Legionella pneumophila*	0.999	0.996	93.595	80.197
	GAPDH	0.997	0.996	98.181	86.813
C	*Chlamydia pneumoniae*	0.996	0.953	104.012	123.528
	*Mycoplasma pneumoniae*	0.998	0.971	94.138	90.427
	*Streptococcus pneumoniae*	0.999	0.999	93.438	93.779
	GAPDH	0.998	0.989	97.66	95.672

### Specificity of the multiplex real-time RT-PCR

**Figure 2:**
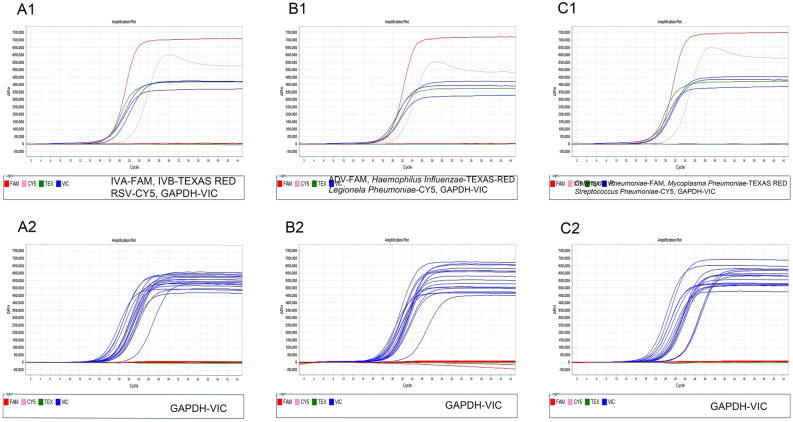
Specificity of multiplex real-time PCR. A1, B1 and C1 show that FAM, TEXAS RED and Cy5 fluorescent signals were generated only by the target pathogens and non-target pathogens were not amplified by the assay, and VIC fluorescent signals were selectively displayed to indicate the internal reference gene GAPDH amplified from the corresponding target pathogens. A2, B2 and C2 show that the VIC fluorescent signals from the DNA of the GAPDH gene for all other existing pathogens were generated by the three-tube real-time PCR system to exclude false negative results.

The analytical specificity of the multiplex real-time RT-PCR assay was evaluated by simultaneously detecting a panel of respiratory infection-associated, non-target microorganisms (listed in Table 7) along with the target pathogens in the same tubes. The data (Fig. 2) indicated that none of the control microorganisms were detected among the target pathogens (A1, B1, C1), whilst the observation of a fluorescence signal from GAPDH, the internal reference gene of each control pathogen, confirmed the presence of genuine nucleic acids in the reaction tubes (A2, B2, C2).

**Figure 3:**
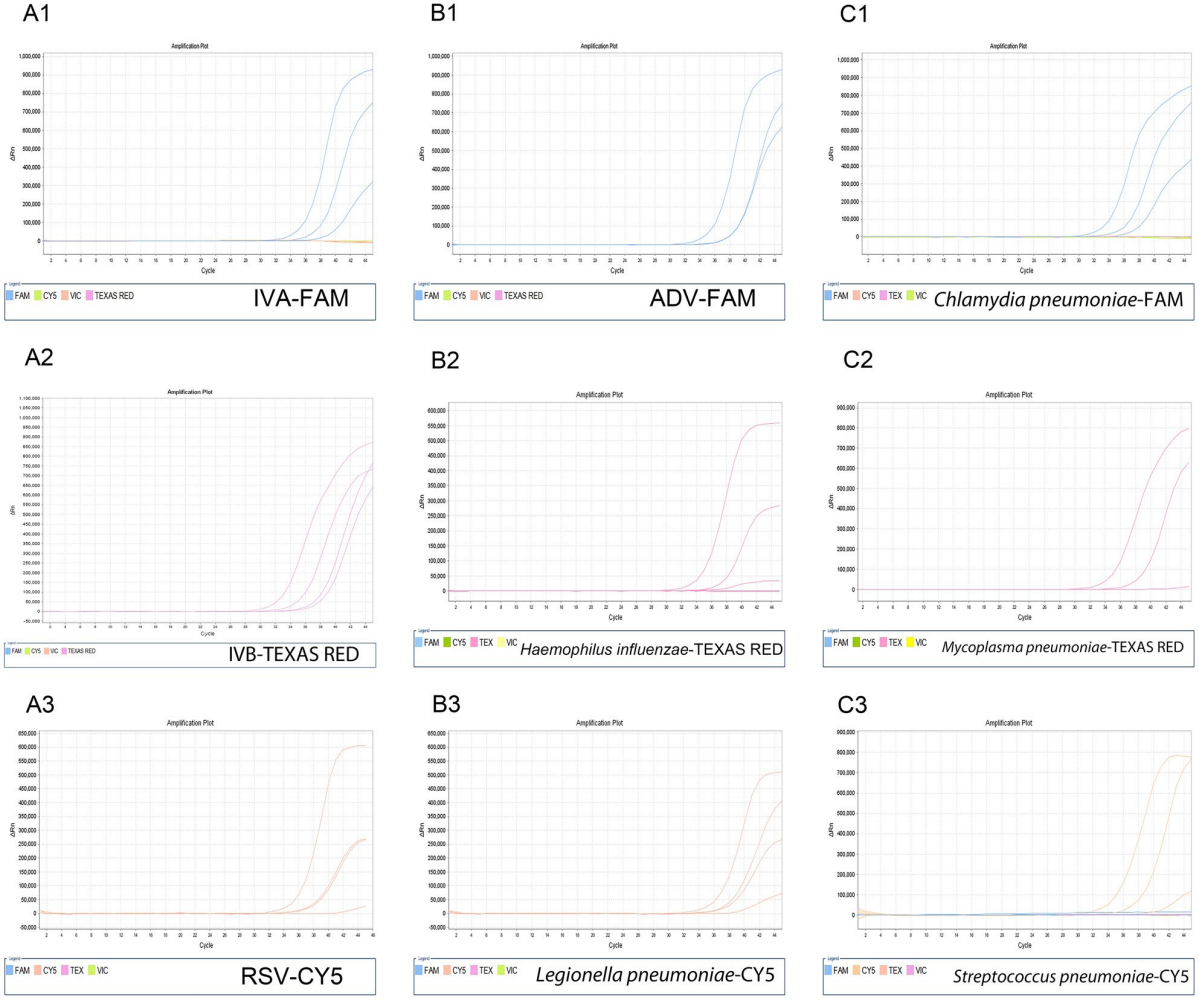
Analytical sensitivity of the multiplex real-time RT-PCR. Two-fold dilutions of the nucleic acids extracted from target pathogens (1000 copies/ml to 125 copies/ml). A1–A3, B1–B3 and C1–C3 are the amplification curves of the corresponding pathogens in tube A, B and C, respectively. Among them, all curves of 500 copies/ml were amplified, and IVA, IVB and *Chlamydia pneumoniae* were undoubtedly detected at 250 copies/ml.

### Detection limits of the multiplex real-time RT-PCR

Since a 100% detection rate of 500 copies/ml of each target gene was confirmed by the corresponding standard curve, serial dilutions of each standard nucleic acids, ranging from 1000, 500, 250 and 125 copies/ml of the template, was subject to detection by multiplex real-time RT-PCR for detection of the assay limit. Figure 3 shows detectable fluorescence signals from each pathogen in the three-tube multiplex real-time PCR (Fig. 3, A1–A3, B1–B3, C1–C3). Twenty repetitions of the lowest detectable concentration of the template confirmed all 9 target genes were stably detected at 500 copies/ml (with detection rates above 90%), with IVA, IVB and Chlamydia pneumoniae being measured at 250 copies/ml. These results indicated that the limits of detection of multiplex real-time RT-PCR were determined to be 250-500 copies/ml, or 1.25–2.5 copies/reaction.

### Precision of the multiplex real-time RT-PCR

As described in the “Methods”, the assay was demonstrated to be highly precise and reproducible by the examination of intra-assay repeatability and inter-assay reproducibility. Intra-assay repeatability was tested in triplicate for each pathogen dilution within the same run; each tested concentration of the template nucleic acid was repeated on three different days to evaluate the inter-assay variability of the multiplex real-time RT-PCR. Three concentration scales, two moderate 10^5^ copies/ml and 10^3^ copies/ml, and a lowest detection limit, as determined above, 500 copies/ml (250 copies/ml for IVA, IVB and *Chlamydia pneumoniae*) were used in these experiments, as shown in Table 2. The intra-assay coefficient of variation (CV) ranged from 0.4 to 1.7%, showing a low Ct value variability when using a concentration of 10^5^ copies/ml for each gene. A slightly increased CV range of 0.4–4.2% was observed when tested at a lower concentration nucleic acid concentration of 10^3^ copies/ml. Interestingly, this trend did not continue with the detection of nucleic acid concentrations at the lowest detection limits, and although the Ct values for each pathogen detected were already close to the cutoff value of 38, a generally consistent CV range of 0.6–3.4% was calculated from data obtained. The inter-assay CV for the Ct values was found to fluctuate from 0.5 to 3.4%; the analytical performance of the assay improved when using the highest concentration of 10^5^ copies/ml of nucleic acids but this performance was not compromised for the worst when testing the lowest detection limit concentrations of nucleic acids.

**Table 2:** Intra-assay and inter-assay of the multiplex real-time RT-PCR.

Target	Concentration (copies/ml)	Intra-assay						Inter-assay					
		Ct of triplicate			Mean	Std	CV%	Mean Ct of three days			Mean of the means	Std	CV%
IVA	10^5^	26.20	26.18	25.78	26.053	0.237	0.9	26.053	26.403	26.813	26.423	0.355	1.3
	10^3^	30.91	32.80	33.16	32.290	1.209	3.7	32.290	33.547	33.883	33.240	1.080	3.2
	250	35.21	35.16	36.03	35.467	0.925	2.6	35.467	35.313	36.125	35.635	0.880	2.5
IVB	10^5^	26.85	26.77	26.06	26.560	0.435	1.6	26.560	26.580	27.337	26.826	0.450	1.7
	10^3^	31.17	33.27	33.57	32.670	1.308	4.0	32.670	33.590	34.107	33.456	0.961	2.9
	250	35.21	35.97	36.92	36.033	1.187	3.3	36.033	35.990	36.600	36.208	1.041	2.9
RSV	10^5^	25.70	25.73	24.98	25.470	0.425	1.7	25.470	25.637	26.387	25.831	0.512	2
	10^3^	30.65	32.94	33.09	32.227	1.367	4.2	32.227	32.893	33.653	32.924	0.956	2.9
	500	33.78	33.8	34.01	33.863	0.197	0.6	33.863	33.757	33.897	33.839	0.166	0.5
Adenovirus	10^5^	26.33	26.34	26.10	26.257	0.136	0.5	26.257	26.450	26.893	26.533	0.317	1.2
	10^3^	33.21	32.69	32.87	32.923	0.264	0.8	32.923	33.367	34.047	33.446	0.582	1.7
	500	34.69	35.43	34.69	34.937	0.397	1.2	34.937	34.603	34.053	34.531	0.511	1.5
*Legionella* *pneumoniae*	10^5^	27.05	26.95	26.74	26.913	0.158	0.6	26.913	26.893	27.370	27.059	0.258	1.0
	10^3^	33.59	33.39	33.33	33.437	0.136	0.4	33.437	33.817	34.423	33.892	0.507	1.5
	500	34.01	34.17	34.32	34.036	0.248	0.7	34.036	33.867	33.877	33.926	0.248	0.7
*Haemophilus* *influenzae*	10^5^	26.96	26.85	26.68	26.830	0.141	0.5	26.830	26.633	27.230	26.898	0.300	1.1
	10^3^	33.62	33.30	33.52	33.480	0.164	0.5	33.480	33.460	34.240	33.727	0.489	1.5
	500	35.04	34.84	35.42	35.100	1.187	3.4	35.100	34.587	34.690	34.792	0.411	1.2
*Mycoplasma* *pneumoniae*	10^5^	27.16	27.00	26.97	27.043	0.102	0.4	27.043	27.387	27.177	27.202	0.368	1.4
	10^3^	34.26	34.71	34.40	34.457	0.230	0.7	34.457	34.977	34.577	34.670	0.537	1.5
	500	34.82	34.46	34.42	34.550	0.263	0.8	34.550	34.287	34.270	34.369	0.612	1.8
*Chlamydia* *pneumoniae*	10^5^	25.41	25.55	25.24	25.400	0.155	0.6	25.400	25.717	26.653	25.923	0.596	2.3
	10^3^	32.09	32.32	32.25	32.220	0.118	0.4	32.220	32.803	33.710	32.911	0.701	2.1
	250	35.42	36.43	36.47	36.107	0.779	2.2	36.107	35.267	35.910	35.761	1.212	3.4
*Streptococcus* *pneumoniae*	10^5^	25.34	25.51	25.18	25.343	0.165	0.7	25.343	25.673	26.717	25.911	0.673	2.6
	10^3^	31.97	32.17	32.17	32.103	0.115	0.4	32.103	32.627	33.807	32.846	0.846	2.6
	500	33.95	34.15	33.89	33.997	0.218	0.7	33.997	33.933	33.657	33.862	0.251	0.7

### Results of the multiplex real-time RT-PCR with clinical samples

**Figure 4:**
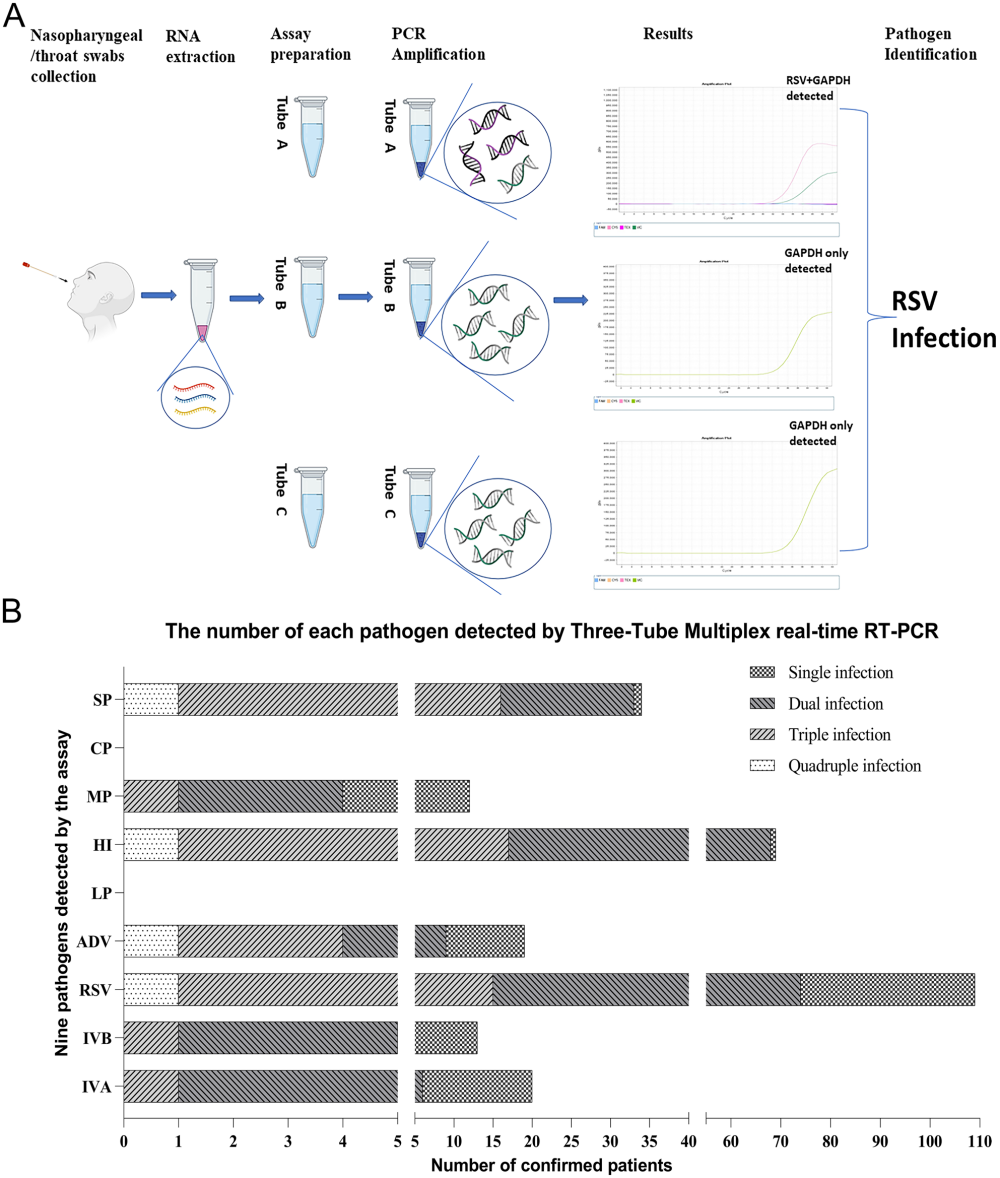
Diagnostic performance evaluation of the multiplex real-time RT-PCR. A, schematic representation of flow chart of the multiplex real-time PCR assay for detection of clinical specimens. After the patient's nasopharyngeal/throat swab is taken, the nucleic acid extracted from the sample is amplified simultaneously in three tubes by the multiplex PCR assay. If the fluorescence channel of RSV in tube A shows an amplification curve and GAPDH is detected in all three tubes, a valid sample collection can be diagnostic for RSV infection in the sample. B, the number of each pathogen detected by three-tube nine-plex real-time PCR. The total number of pathogens detected in each type of infection were as follows: Single, 7; Dual, 7; Triple, 7; Quadruple, 4. LP: *Legionella pneumophila*, HI: *Haemophilus influenzae*, MP: *Mycoplasma pneumoniae*, CP: *Chlamydia pneumoniae*, SP: *Streptococcus pneumoniae*.

Multiplex real-time PCR assay for 179 nasopharyngeal/throat swabs detected 167 (93.30%) specimen with at least one positive infection. Among them, 109 (60.89%) RSV infections were found to be the predominant causative agent in the studied patient cohort, with 17 (9.50%) IVA infections, 20 (11.17%) IVB cases and 19 (10.61%) ADV-positive patients constituting much smaller subgroups of medical cases of viral-infection. With the designed capacity for detecting several common bacteria, this multiplex real-time RT-PCR assay identified at least one type of bacterial infection in 99 (55.31%) specimens. When excluding the number of cases with concomitant viral infections, the assay detected 10 (5.59%) cases with single bacterial infection. Furthermore, the results revealed that virus-bacteria co-infections were found in 90 (50.28%) patients. The complexity of this virus-bacteria co-infections can be subdivided into 72 cases of dual infections, including 43 cases of RSV combined with *Haemophilus influenzae*, 14 cases of RSV combined with *Streptococcus pneumoniae*, 3 cases of IVA combined with *Haemophilus influenzae*, 2 cases of ADV combined with *Haemophilus influenzae*, 1 case of each RSV and *Mycoplasma pneumoniae*, IVA and IVB, IVA and *Streptococcus pneumoniae*, IVB and *Streptococcus pneumoniae*, IVB and *Mycoplasma pneumoniae*, IVB and *Haemophilus influenzae*, ADV and *Streptococcus pneumoniae*, ADV and *Mycoplasma pneumoniae*, ADV and *Haemophilus influenzae*, RSV and *Haemophilus influenzae*; 17 cases of triple infections, including 13 cases of RSV combined with *Haemophilus influenzae* and *Streptococcus pneumoniae*, 1 case of each IVA combined with *Haemophilus influenzae* and *Streptococcus pneumoniae*, IVB and ADV combined with *Mycoplasma pneumoniae*, ADV combined with *Haemophilus influenzae* and *Streptococcus pneumoniae*, RSV and ADV combined with *Haemophilus influenzae*; and 1 case of quadruple infections which was determined as RSV and ADV combined with *Haemophilus influenzae* and *Streptococcus pneumoniae*. Figure 4 shows a schematic illustration of the workflow of multiplex real-time PCR assay for detection of clinical specimens and the number of pathogens detected by three-tube multiplex real-time RT PCR in 179 clinical specimens, including duplex, triplex, and quadruplex co-infections. Whereas the immunofluorescence assay performed by the central lab of the hospital reported a total of 157 (87.71%) specimens with viral infections, including 110 (61.45%) RSV infections, 21 (11.73%) IVA-positive cases, 11 (6.15%) IVB-confirmed patients and 15 ADV (8.38%) infections. Table 3 summarized the comparisons between the results of the two assays. Notably, three Immunofluorescence assay-positive specimens were reported as negative by multiplex real-time RT PCR, including 2 RSV and 1 IVA. These three cases were confirmed as true negative by sequencing, resulting in the sensitivities of multiplex real-time RT-PCR for RSV and IVA both 100%. Conversely, four specimens that tested as negative for viral infection by the Immunofluorescence assay were detected as positive by the multiplex real-time RT-PCR method, including one RSV and three ADV; they were either single virus infections or co-infections with bacteria. Again, sequencing confirmed these were true positive for the results of the detected pathogens by the multiplex real-time PCR (shown in Table 4). Cohen’s kappa was evaluated to assess the pairwise agreement between multiplex real-time RT-PCR and Immunofluorescence assay in the detection of viral pathogens, and the coefficient of 0.823 (p = 0.000, 95% confidence interval 0.696–0.951) indicated an almost perfect concordance between the two tests. Among the samples with single or multiple infections reported by multiplex real-time PCR, validation results by Sanger sequencing confirmed the identity of the pathogen samples detected by PCR.

**Table 3:**
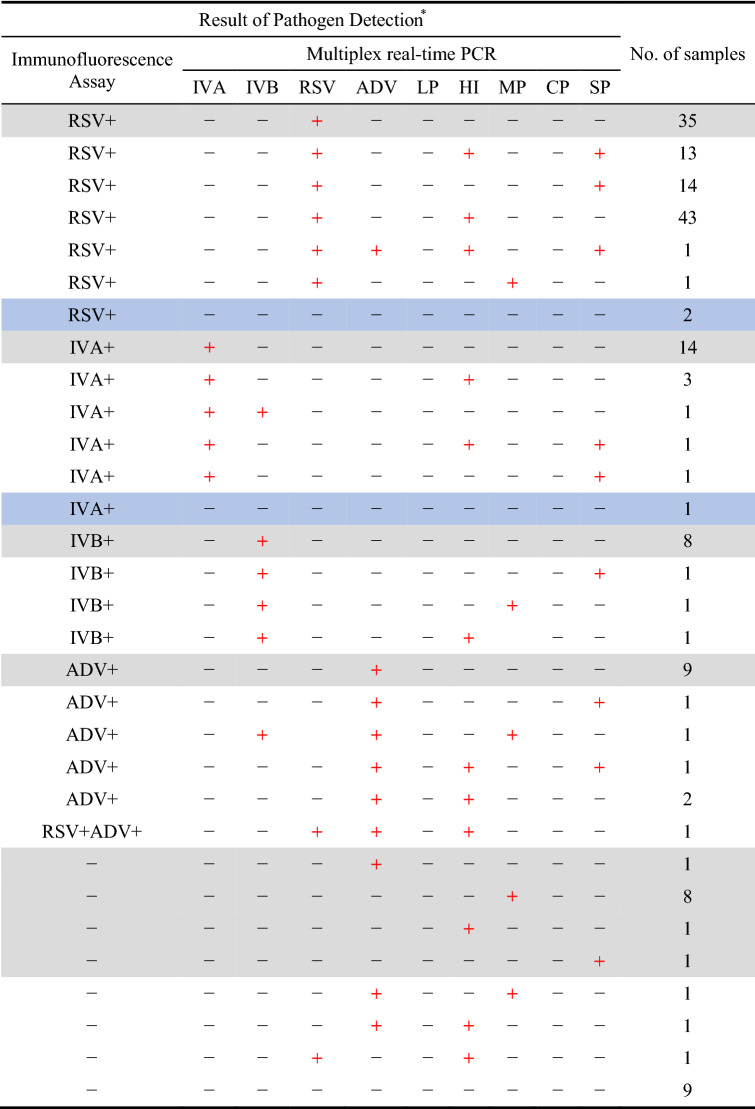
Comparison of multiplex real-time RT-PCR and immunofluorescence assays for detecting 179 clinical specimens.

+, positive; −, negative. LP: *Legionella pneumophila*, HI: *Haemophilus influenzae*, MP: *Mycoplasma pneumoniae*, CP: *Chlamydia pneumoniae*, SP: *Streptococcus pneumoniae*. Grey fill: Single infection confirmed by multiplex real-time RT-PCR; Blue fill: Immunofluorescence Assay reported positive but negative by multiplex real-time PCR.

**Table 4:** Confirmation of Multiplex real-time PCR discordant specimens; pathogens detectable by both multiplex real-time PCR and immunofluorescence assays.

Pathogen*	Result immunofluorescence assay vs multiplex real-time PCR	No	No. confirmed by DNA sequencing
RSV	Immunofluorescence assay^+^ /multiplex real-time PCR^−^	2	0
IVA	Immunofluorescence assay^+^ /multiplex real-time PCR^−^	1	0
ADV	Immunofluorescence assay^−^ /multiplex real-time PCR^+^	1	1
ADV + MP	Immunofluorescence assay^−^ /multiplex real-time PCR^+^	1	1
ADV + HI	Immunofluorescence assay^−^ /multiplex real-time PCR^+^	1	1
RSV + HI	Immunofluorescence assay^−^ /multiplex real-time PCR^+^	1	1

*HI: *Haemophilus influenzae*, MP: *Mycoplasma pneumoniae.*

## Discussion

Acute respiratory infection induced morbidities becomes more serious, and the rate of mortality increases in the elderly and children under 5 years of age^1,10,26^. Considering this, acute respiratory infections increased patient morbidity, hospitalization rates and mortality^3,4^. Acute respiratory infections are caused by a complex array of pathogens, most commonly viruses and bacteria, as well as microorganisms such as *Mycoplasma* and *Chlamydia*^15,18^. Furthermore, clinical diagnosis is complicated with co-infection of several pathogens leading to acute respiratory infection^24^. Thus, the development of a comprehensive, easy to implement and rapid molecular diagnostic tool capable of detecting and distinguishing various types of causative pathogens in clinical samples, is invaluable for epidemiological surveillance, progress prediction and therapeutic strategies selection^2,27,28^. With its sensitivity and specificity regarding pathogen detection, RT-PCR has become a promising tool when looking to perform the rapid screening and identification of multiple pathogens simultaneously^22,23^. This method targets pathogen-specific genetic material, rather than viral or bacterial antigens or antibodies, thus outperforming other traditional chemical immunoassay-based test procedures. Multiplex RT-PCR assays display a variety of benefits, including a significant reduction in the turnaround time of the assay compared to the use of multiple assays^24^. A multiplex RT-PCR method for detecting nine distinct respiratory microbial pathogens simultaneously was first disclosed in 1999^23^; despite being pioneering at the time, this was replaced by TaqMan-based fluorescent probe techniques due to the limitations of conventional PCR, which required post-PCR modifications and lacked sensitivity and quantifiability^21^. In recent years, there has been a rapid growth of commercial and independent laboratory-developed, sequence-specific, amplification-based, multiplex real-time PCR assays^25,28–30^; these are available for the more efficient detection of selected respiratory pathogens^31^.

In the present study, a multiplex real-time RT-PCR assay based on TaqMan probe technology was innovatively developed, standardized and comprehensively validated to achieve the simultaneous and rapid identification of nine selected pathogens; these included common viral and bacterial pathogens frequently found to cause acute respiratory infections in clinical practice^25,31^. The primer–probe pairs used for the assay were carefully designed to optimize amplification, in which elements such as homology of the primers with their target nucleic acid sequences, the G + C content, melting temperature and product length were theoretically optimized. In addition, one of the innovative methodological features of this study was the application of a digital droplet PCR technique to achieve accurate quantification of pathogenic nucleic acid molecules used to construct standard curves, which in turn facilitated the validation of linearity and calculation of a reliable minimum detection limits for this multiplex real-time RT-PCR system. The assay is specific and sensitive for the identification of influenza A and B viruses, adenovirus, RSV, *Streptococcus pneumoniae*, *Legionella pneumophila*, *Haemophilus influenzae*, *Chlamydia pneumoniae*, and *Mycoplasma pneumoniae.* Results demonstrate the multiplex TaqMan RT-PCR was capable of achieving a sensitive detection for the selected nine pathogens in the range of 250–500 copies/ml, equivalent to 1.25–2.5 copies/reaction. With the detection limit of RSV being 2.5 copies/reaction, this assay proves to be more sensitive in the detection of the pathogen than some previously reported multiplex real-time PCR assays either using commercial kits or a laboratory developed nested PCR-based approach^25,26^. However, in this study, considering that the immunofluorescence assay is a qualitative test, quantification of pathogens in copies/ml conducted using standard curves was not used to interpret the results. Therefore, differences in the quantification of a specific target between single pathogen and multi-pathogen infections were not displayed here. There was no amplification from any of several other pathogens including: human metapneumovirus, coronavirus, norovirus, varicella-zoster virus, human rhinovirus, *Staphylococcus aureus*, *Streptococcus pyogenes, Tuberculosis* and *Chlamydia trachomatis* indicating the specificity of the assay*.*

179 clinical samples were utilised for the purposes of further evaluation of the multiplex real-time RT-PCR and comparison with direct immunofluorescence assay, which has been commonly used as a fast-screening tool for suspected respiratory infections in many clinical laboratories. As described in the “Results” section, the pathogen with the largest number of positive infected samples detected by both methods was RSV, with 109 (60.89%) cases detected by multiplex real-time RT-PCR and 110 (61.45%) specimens confirmed by the immunofluorescence assay, respectively. The kappa value of the two detection assays was calculated as 0.823 (*p* = 0.000), indicating an exceptional consistency between the two methods, with regards of viral identification. One of the objectives of this study was to assess whether the multiplex real-time PCR assay that was developed is capable of being a rapid screening tool in a clinical setting. Therefore, nucleotide sequencing for verifying pathogenic sequences in each clinical sample was used as a reference method to validate what was detected by the PCR assay. In contrast with the direct immunofluorescence assay, which is designed to detect 7 common viruses, a significant characteristic of the novel multiplex real-time RT-PCR is its coverage of not only 4 viruses but also 5 types of bacteria, mycoplasma, and chlamydia. Such design of targeting various pathogens enables the assay’s competence to provide comprehensive information with diagnostic value as per the current circumstance that mixed viral-bacterial infections has not yet been well studied. This fully validated multiplex RT-PCR assay based on TaqMan primer–probe technology ensures simultaneous detection and differentiation of the aforementioned respiratory pathogens*.* The specific and sensitive detection revealed that, of 167 positive samples, 99 (55.31%) cases were identified as being bacterial-associated infection, including one patient with quadruple infections detected as RSV and ADV combined with *Haemophilus influenzae* and *Streptococcus pneumoniae*. Such a finding constitutes strong evidence to support that hospitalized patients with acute respiratory infections are likely to be infected by more than one pathogen, while some previous studies reported a dual viral infection rate of approximately 20%^15,27,32,33^. In general, RSV combined with *Haemophilus influenzae* accounted for 43 cases, the highest number of confirmed mixed infections, which is in line with evidence provided by independent studies^2,10,28,34^. Furthermore, apart from a single infection, *Streptococcus pneumoniae* infection was detected in 33 patient samples, making it the second most common bacterium causing acute respiratory infections in this study. There is no doubt that this nine-plex real-time RT-PCR assay will significantly expand the diagnostic potential for a careful evaluation of concomitant bacterial infection to patients with positive results of viral examination, thus help clinicians to adopt rapid and accurate antibiotic treatment regimens.

The nine-plex real-time RT-PCR was not designed from the perspective of a single pathogenic virus or bacterium, but for the rapid identification of a causative pathogen from the most commonly found causative agents of respiratory infections. The significance of this is that it allows for the targeted, time-effective treatment of the infection and minimizes the misuse of antibiotics in vulnerable patients. This reduces the risk of hospitalization and mortality, reducing the burden on the health system and improving patient care^10^. Following a thorough examination of the literature, this study appears to be the first reported trial of a one-step multiplex PCR reaction system capable of identifying multiple respiratory pathogens, comprising DNA and RNA based viruses as well as bacteria, *Chlamydia* and *Mycoplasma,* with a total turnaround time of 3.0 h. In a single tube, this study designed four different sets of primers-probe for four target genes, inclusive of one reference gene, and achieved quadruple detection, actualizing a total of ten genes identification in a system of three-tube on a four-channel fluorescent PCR instrument. To minimize the probability of false-negative results, GAPDH was exploited to verify that sufficient amounts of nucleic acids was extracted from adequate scraping of the mucosal surface. Furthermore, a mixture of the reference viral and bacterial strain extracts with concentrations determined by the digital droplet PCR was utilised as an internal control. This ensures maximum compatibility of this nine-plex real-time PCR assay to simultaneously detect the selected pathogens on mainstream fluorescent PCR instrument models. In addition to its broad applicability, the affordability of the assay is another significant advantage. The cost per sample was approximately $20, half of the cost of single plex real-time RT-PCR assays for detection of the same nine pathogens if conducting nine individual experiments. Such economy makes our assay advantageous compared to commercially available multipathogen screening panels that require specialized instruments to run, such as Luminex’s NxTAG respiratory pathogen panel, the FilmArray respiratory panel and Panther Fusion respiratory assay. Furthermore, with the closed one-tube design, this assay is competent to minimise the likelihood of cross-contamination whilst enabling sensitive and specific detection of co-infection.

The original aim of this present study was to demonstrate the establishment, optimization, and evaluation of the novel multiplex real-time RT-PCR, to this regard, demographic characteristics of the studied patients were not included herein. Further, the data analysis related to detected agents was not further expanded to more clinical aspects, such as positive outcomes grouped by age, clinical severity, duration of symptoms, and length of hospitalization. Hence, a detailed explanation as to why RSV was reported as the most prevalent respiratory agent in this study is absent, but unreported data from a majority of paediatric cases and most of the specimens collected during Winter and Spring seasons supplied by clinical collaborators in this study all support our findings were not obviously distinct from the literature. In fact, a study analyzing nasopharyngeal swab specimens that were collected from paediatric inpatients in Shenzhen Children’s hospital within the same region between May 2009 and April 2010 reported that RSV was detected most frequently in infected cases among all selected patients by using a QIAGEN's multiplex ResPlex real-time PCR kit^26^. Given the similarities between our study and the previous report in aspects including patient age, seasons of admission, specimen collection method and pathogen detection technique are supportive perspectives validating our results showing a high frequency of RSV. Likewise, the results of quantifying the infected pathogen load in relevant samples with detected Ct values, despite being of great interest, are not presented herein, due to the lack of correlation with corresponding clinical data. Further analysis of these data is required. In addition, as a methodological comparison, this study was limited by the comparison with the results of a direct immunofluorescence assay performed by laboratory technicians in the collaborative hospital from patient nasopharyngeal/throat swab samples, with the lack of the obtainment of single-plex real-time PCR data using commercially established assays due to limited volume of the starting samples being the primary limitation of this study. Other limitations include the absence of an additional introduction of other clinical laboratory data, such as pathogen cultures and antigen and antibody-specific serologic assays. Whether nasopharyngeal/throat swabs are the appropriate specimen for suspected infections also needs to be addressed. The authors recommend further sputum culture of *Haemophilus* *influenzae* and *Streptococcus* *pneumoniae* if identified by this multiplex PCR, given the requirement to differentiate between nasopharyngeal colonization and pulmonary infection^35^. However, this was not possible in this study due to simultaneous running of the assay with clinical sample collection. Besides, DNA quantities determined by real-time PCR are usually associated with bacterial load, which could indicate where there is a “real” infection. Future work including further evaluation of the multiplex real-time RT-PCR utilising larger number of clinical samples and integrating more clinical data to enable a comprehensive analysis will provide beneficial information to help clinicians in the aspects of therapeutic strategies and prognosis prediction.

## Conclusion

It was demonstrated that this innovatively developed multiplex real-time RT-PCR assay possesses excellent performance characteristics, with the potential to provide clinicians detailed genetic information of causative pathogens. This assay could be utilised for rapid diagnosis acquisition leading to the prompt initiation of treatment, which may have a substantial positive impact on patient outcome. Establishing this assay enabled the simultaneous monitoring of influenza A and B viruses, adenovirus, RSV, *Streptococcus pneumoniae*, *Legionella pneumophila*, *Haemophilus influenzae*, *Chlamydia pneumoniae*, and *Mycoplasma pneumoniae*. The assay presented herein exhibited improvements to sensitivity, specificity and time and cost-effectiveness compared to previous assays utilised for clinical and epidemiological applications.

## Materials and methods

### Ethical approval

This study was institutionally approved by Guangdong Pharmaceutical University, the Medical Ethics Committee of the First Affiliated Hospital of the University, and the Medicine and Biological Engineering Technology Research Center of the Ministry of Health, Guangzhou, China, in correspondence with the guidelines of the collection of patient specimens and the use of human biological samples for scientific analysis, as well as data acquisition and presentation. Informed written consent was provided by the participants (or parents, in the case of a minor) involved in the study. In accordance with the ethical requirements, the analysis of all clinical samples was performed in an anonymized manner. All experimental protocols were carried out in accordance with the relevant guidelines and regulations.

### Viral and bacterial strains

A total of 25 viral and bacterial strains, including diverse subtypes of the target respiratory viral and bacterial pathogens, used as positive controls for the optimization of multiplex PCR and validation of the sensitivity of this real-time PCR assay, were obtained-listed in Table 5. All pathogenic microorganisms were stored at − 80 °C until DNA/RNA were extracted for analysis.

**Table 5:** Pathogenic microorganisms, including culture strains and laboratory isolates, used as the target pathogens in the evaluation of three-tube multiplex real-time RT-PCR.

Number	Species	Subtypes	Strain ID	Supplier	Description
1	Influenza A virus	H1N1	ATCC VR95	American Type Culture Collection	
2		H3N2	GDV124	The State Key Laboratory of Virology, Wuhan University, CHINA	
3		H1N1 2009	GDV108		
4		H5N1		The National Institute for Food and Drug Control, CHINA	Supplied as virus cultures
5		H7N9			
6	Influenza B virus	IVB Yamataga	GDV105	The State Key Laboratory of Virology, Wuhan University, CHINA	
7		IVB Victoria	GDV104		
8	Respiratory syncytial virus	RSV A	ATCC VR1540	American Type Culture Collection	
9		RSV B	ATCC VR 955		
10	Adenovirus	Human adenovirus 1	ATCC VR-1	American Type Culture Collection	
11		Human adenovirus 2	ATCC VR-846		
12		Human adenovirus 3	ATCC VR-3		
13		Human adenovirus 4	ATCC VR-1572		
14		Human adenovirus 5	ATCC VR-5		
15		Human adenovirus 46	ATCC VR-1308		
16		Human adenovirus 48	ATCC VR-1406		
17		Human mastadenovirus 7	ATCC VR-7		
18		Human adenovirus 55	N/A	The State Key Laboratory of Virology, Wuhan University, CHINA	Supplied as clinical isolates
19	*Chlamydia pneumoniae*		ATCC VR2282	American Type Culture Collection	
20	*Mycoplasma pneumoniae*	*Mycoplasma pneumoniae* Somerson et al	ATCC 15531		
21	*Streptococcus pneumoniae*	*Streptococcus pneumoniae* Serotype 19F	ATCC 49619		
22		*Streptococcus pneumoniae* (Klein) Chester	ATCC 700669		
23	*Legionella pneumoniae*	*Legionella pneumophila* subsp. *fraseri* Brenner et al	ATCC 33156		
24		*Legionella pneumophila* subsp. *pneumophila* Brenner et al	ATCC 43290		
25	*Haemophilus influenzae*	*Haemophilus influenzae* (Lehmann and Neumann) Winslow et al	ATCC 10211		

### Clinical specimens

Nasopharyngeal/throat swabs were collected in universal transport medium (Supplied by Diagnostic Hybrids, USA) from 179 patients primarily diagnosed with acute respiratory infections. Samples were collected from the respiratory ward of the First Affiliated Hospital of Guangdong Pharmaceutical University within the first 24 h of each patient admission between November 2017 and July 2019. All patients exhibited clinical manifestations of acute respiratory infections with at least two of the following symptoms: cough, pain, soar throat, expectoration and tachypnoea accompanied by fever (temperature > 38 °C). The specimens were collected by specialized professionals and sent to the central laboratory of the hospital for microbiological and immunological analysis. 500 µl aliquots of nasopharyngeal/throat swab samples were sent to our laboratory for the use in the validation of the multiplex real-time RT-PCR.

### DNA and RNA extraction

The DNA from 9 subtypes of adenovirus and 7 bacteria and the RNA from a total of 9 subtypes of the target viruses, were extracted using a Nucleic Acid Isolation Kit and a Smart32 Nucleic Acid Extraction instrument, (both supplied by Da’an Gene, Guangzhou, China) in accordance with the manufacturer’s instructions.

The clinical nasopharyngeal/throat swab samples were 500 µl; 200 µl of the mixture was aliquoted for nucleic acid extraction using the same total nucleic acid kit and nucleic acid extraction instrument from Da’an Gene, in line with the corresponding section of the manufacturer’s handbook. The DNA was eluted in a final volume of 70 µl and stored at − 80 °C.

### Design of primer–probe pairs for multiplex real-time RT-PCR

Ten sets of primer–probe designed for multiplex real-time RT-PCR are specified in Table 6. Specific primers and probes for the selected targets, nine pathogens plus one internal reference, were designed based on DNA sequences from the International Nucleotide Sequence Database Collaboration (INSDC) at the National Center for Biotechnology Information (NCBI), by utilising the oligo primer analysis software Oligo7 (http://oligo.net). A single gene was chosen from each pathogen to be the target of PCR amplification. The primer and probe design principal for the multiplex real-time RT-PCR assay followed the pattern of selecting conserved gene sequence regions. Validating tests were conducted including testing of specificity using: Basic Local Alignment Search Tool (BLAST, http://blast.ncbi.nlm.gov/Blast.cgi), the evaluation of the hairpin of internal primers, primer-dimer potential, G-C content and the melting temperatures of the primers and probes by Oligo7, and sequence comparison analysis by Bioedit software (https://bioedit.software.informer.com/). The expected amplicon sizes were between 83 and 127 bp. Primer melting temperature ranged from 55 to 65 °C. TaqMan probes used to determine subgroup specific amplification in each reaction were individually labelled diverse report dyes including FAM, Texas Red, CY5, and VIC on the 5′ end and quencher dyes MGB and BHQ2 on the 3′ end, to differentiate the different pathogens in the same reaction system. All primers and probes were synthesized by Da’an Gene (Guangzhou, China).

**Table 6:** Primers and probes used in the multiplex real-time RT-PCR.

Pathogen	Target gene	Sequence (5′ to 3′; with fluorescent reporter dyes for individual probe)	Working concentration (pmol/µl)	Amplicon sizes (bp)
Influenza A virus	Matrix protein 1	F: GGAATGGCTAAAGACAAGACCAAT R: CATTTTGGACAAAGCGTCTACG P: 5′FAM-CCTCGCTCACTGGGCACGGT-3′MGB	0.24	119
			0.24	
			0.08	
Influenza B virus	Matrix protein 1	F: GGAGAAGGCAAAGCAGA R: CATTCCAAGGCAGAGTCTA P: 5′Texas Red-ACTGTTGGTTYGGTGGGAAA-3′MGB	0.32	83
			0.32	
			0.16	
RSV	Nucleoprotein	F1: TGGCTCTTAGCAAAGTCAAGTTA R1: TGTCAATATTATCTCCTGTACTACGTT P1: 5′CY5-CATTAAATAAGGATCAGCTGCTGTCATCC-3′BHQ2	0.4	82
			0.4	
			0.16	
		F2: GCTCTTAGCAAAGTCAAGTTGAA R2: TGCTCCGTTGGATGGTGTAT P2: 5′CY5-CTCAACAAAGATCAACTTCTGTCATCCAG-3′BHQ2	0.4	
			0.4	
			0.16	
Adenovirus	Hexon	F: GTAGACTTGCARGACAGAAACAC	0.25	126
		R1: AAYRCGAACATCRGGATCRTAAC	0.25	
		R2: TGATTCTAACATCDGGATCATAGC	0.25	
		P: 5′FAM-ATGTGGAABCAGGCTGTTGAC-3′MGB	0.2	
*Legionella pneumoniae*	Mip	F: GGTGCCGATTTGGGGAAGA R: TGAGCGCCACTCATAGCGTC P: 5′CY5-CATGCCTTTAGCCATTGCTTCCG-3′MGB	0.2	95
			0.2	
			0.1	
*Haemophilus influenzae*	OmpP6	F: CCAGTAATGTCGTATTTATCAA R: GTTCCTCTAACAACGATGC P: 5′Texas Red-TCAGCAACAGAGTATCCGCCAAAAGT-3′BHQ2	0.15	124
			0.15	
			0.05	
*Mycoplasma pneumoniae*	P1 cytadhesin RepMP4	F: GCAACCGTACCGCCATT R: TCAGTCCCACAAACCGTG P: 5′Texas Red-CGAACTATCCGCCCAGTTGAAGAAC-3′MGB	0.15	113
			0.15	
			0.075	
*Chlamydia pneumoniae*	Outer membrane protein A	F: CAAAGTCTGCGACCATCAAT R: TACTCCAATGTATGGCACTAAAG P: 5′FAM-TGAATGGCAAGTAGGAGCCTCT-3′MGB	0.15	89
			0.15	
			0.075	
*Streptococcus pneumoniae*	LytA	F: CACCTTCTTCGTTGAAATAGT R: TGGAGGAAGCACACAGAC P: 5′CY5-AGCCATTTCGCCTGAGTTGTCG-3′MGB	0.25	109
			0.25	
			0.125	
GAPDH	Glyceraldehyde-3-phosphate dehydrogenase	F: TGCCTCTTGTCTCTTAGATTTGG R: TGATGGCAACAATATCCACTTTACC P: 5′VIC-TCACCAGGGCTGCTTTTAACTC-3′MGB	0.15	88
			0.15	
			0.075	

### Establishment of the multiplex real-time RT-PCR assay

A three-tube, four-plex, one-step RT-PCR system was developed to accommodate the simultaneous detection of the selected nine pathogens. The pathogen subgroups were designed as follows: tube A: IVA, IVB, and RSV; tube B: adenovirus, *Haemophilus influenzae*, and *Legionella pneumophila* and tube C: *Chlamydia pneumoniae*, *Mycoplasma pneumoniae*, and *Streptococcus pneumoniae*. In the initiation stage, the nine sets of primer–probe pairs targeting the selected pathogens were validated using single plex PCR reactions to confirm the optimized conditions (annealing time/temperature, primer, and probe concentrations). In addition to the pathogen specific sets of primer–probe pairs, each tube of the three-tube system contained a primer–probe pair for GAPDH, to serve as an internal reference gene. The multiplex real-time PCRs were performed in 25 µl of a reaction mixture which comprised of: primer concentrations ranging from 2 to 10 pmol, fluorescent probe concentrations ranging from 5 to 10 pmol, a 6 U hot start Taq enzyme, 20 U RNasin (supplied by Da’an Gene, China), 10 U MMLV (Fapon Biotech, Shenzhen, China), 75 nmol Mg^2+^, 1.5 µmol Tris–HCl (pH 8.8), 0.25 µmol (NH4)_2_SO_4_, 1.25 µmol KCl, (all from Sigma-Aldrich, Germany), 0.875 µmol deoxynucleotide triphosphates (A:C:G:T = 1:1:1:1, supplied by Promega, USA), 5 µl template DNA or RNA and diethyl pyrocarbonate-treated water. An ABI 7500 Real-Time PCR system (Thermo Fisher Scientific, USA) was used for the experiment. RT was performed at 50 °C for 15 min, followed by 15 min at 95 °C, this was followed by 45 cycles of 15 s at 94 °C and 45 s at 55 °C, which amplified the cDNA and DNA. Fluorescent signals were recorded during the annealing phase of the 45 cycles. Cycle threshold (Ct) values were determined by the 7500 Real-Time PCR software at the automatic threshold setting with the Ct value of 38 chosen as a cut-off value for defining positivity. Each multiplex PCR run consisted of one negative control and one positive control. The positive control consisted of a mixture of nucleic acids of known concentration extracted from the reference viral and bacterial strains. All PCR-relevant procedures were carried out in designated PCR suites, with separate processes in different rooms, running routine decontamination. The total experimental turnaround time, from the initiation of nucleic acid extraction to the completion of target genes fluorescent signal production, was around 3.0 h.

### The absolute quantification of nucleic acids by droplet digital PCR

A droplet digital PCR (ddPCR) technique was utilised to directly and precisely quantify the copy numbers of nucleic acids extracted from viral and bacterial cultures. Herein, a One-Step ddPCR Advanced kit for Probes (Bio-Rad Laboratories, Hercules, CA) was used in accordance with the manufacturer’s recommendation and as previously described^36^. Briefly, the reaction mixtures were assembled with 5 µl ddPCR supermix, 40 U Reverse Transcriptase, 1 µl 300 mM Dithiothreitol (DTT), the same TaqMan primers and probes used in the multiplex real-time RT-PCR (final concentration of 500 and 250 nM, respectively), and 5 µl template nucleic acids in a final volume of 20 µl. Each reaction was loaded into the sample well of an eight-well droplet cartridge together with 70 µl of droplet generation oil (Bio-Rad). Following their formation in a QX200 droplet generator (Bio-Rad), droplets were then transferred to a 96-well PCR plate, which was heat-sealed with foil before, amplification was performed using a C1000 Touch Thermal Cycler (Bio-Rad) with the following RT-PCR parameters: a reverse transcription step at 50 °C for 60 min; initial denaturation at 95 °C for 10 min, followed by 45 cycles of 94 °C for 30 s and 55 °C for 1 min; and a final extension step at 98 °C for 10 min. The PCR plate was subsequently scanned on a QX200 droplet reader (Bio-Rad) and the copies/µl of each queried target per well were analysed with QuantaSoft software version 1.7 (Bio-Rad). Droplet positivity was determined by fluorescence intensity and only droplets above a minimum threshold of fluorescence amplitude were judged as positive. By utilising ddPCR, the concentrations in copy/ml of the reference viral and bacterial strains used for the establishment of standard curves were determined. All experiments were performed in triplicate.

### The evaluation of sensitivity and specificity

Twenty-five strains, namely: H1N1, H1N1 (2009), H3N2, H5N1, H7N9, IVB Yamagata, IVB Victoria, RSV (types A and B), *Haemophilus influenzae*, *Legionella pneumoniae* (subsp. *fraseri* Brenner et al. and subsp. *pneumophila* Brenner et al.), *Streptococcus pneumoniae* (Serotype 19F and Klein Chester), *Mycoplasma pneumoniae*, *Chlamydia pneumoniae*, and adenovirus (types 1, 2, 3, 4, 5, 7, 46, 48 and 55), were used to determine the analytical sensitivity of our assay. Serial ten-fold dilutions of the 25-target pathogenic DNA/RNA samples with known starting concentrations determined by ddPCR were subject to detection by the multiplex TaqMan RT-PCR assay, to determine the assay linearity and limits of detection. The Ct values obtained from the serial dilutions were graphed on the Y axis against the log of the dilution on the X axis, to generate standard curves for each target gene. The slope of each standard curve was calculated in accordance with the equation: E = 10^(−1/slope)^ − 1, to determine the efficiency (E). The additional viral and bacterial species unrelated to the target pathogens, used to evaluate the specificity of the multiplex TaqMan RT-PCR assay, are shown in Table 7. A mixture of undiluted nucleic acid from each of these microorganisms was simultaneously tested alongside the target nucleic acid to assess the absence of non-specific amplification.

**Table 7:** Pathogenic microorganisms, including culture strains and laboratory isolates, used as the control pathogens in evaluation of the three-tube multiplex real-time RT-PCR.

Number	Species	Subtypes	Strain ID	Supplier	Description
1	Human metapneumovirus			The State Key Laboratory of Virology, Wuhan University, CHINA	Supplied as clinical isolates
2	Human coronavirus	229E			
3		HKU1			
4		OC43			
5		NL63			
6	Norovirus				
7	Rotavirus				
8	Cytomegalovirus				
9	Human metapneumovirus				
10	Mumps orthorubulavirus			The National Institute for Food and Drug Control, CHINA	Supplied as virus or bacteria cultures
11	Varicella-zoster virus				
12	Parainfluenza virus	type 2			
13	*Bordetella pertussis*				
14	*Staphylococcus aureus*				
15	*Streptococcus pyogenes*				
16	*Klebsiella pneumoniae*				
17	*Mycobacterium tuberculosis*				
18	Human rhinovirus	type 7	ATCC VR-1601	American Type Culture Collection	
19		type 79	ATCC VR-1189		
20	*Chlamydia trachomatis*	type A Har-13	ATCC VR-571B		
21		type B Har-36	ATCC VR-573		
22		type C TW-3	ATCC VR-1477		
23		type D UW-3	ATCC VR-885		
24		type E Bour	ATCC VR-348B		
25		type F IC-Cal-3	ATCC VR-346		

### The validation of reproducibility

Intra-assay reproducibility tests were carried out in triplicate by respectively testing three different concentration mixtures (10^5^ and 10^3^ copies/ml and a lowest limit of detection, either 500 or 250 copies/ml) of each pathogenic nucleic acid within the same experiment. The inter-assay variability was examined by repeating the intra-assay run on three continuous days to validate the reproducibility of the multiplex real-time RT-PCR.

### Clinical sample verification

Nucleic acids extracted from aliquots of the 179 clinical nasopharyngeal/throat swab specimens were tested using the multiplex real-time RT-PCR. The viral results were compared with conventional immunofluorescence assay results that employed a D^3^ Ultra DFA Respiratory Virus Screen and ID kit. These kits used fluorescein-conjugated monoclonal antibodies that targeted the viral antigens of IVA, IVB, parainfluenza virus types 1, 2 and 3, RSV and adenovirus (Supplied by Diagnostic Hybrids, USA) (performed by the central laboratory of the collaborative hospital). Nucleic acids from all 179 samples were further analysed by Sanger sequencing which was designed as the reference method in this study. The samples in which the PCR data was inconsistent with the immunofluorescence results were confirmed by gene sequencing. Furthermore, the possibility that the pathogenic microorganisms carried in the clinical specimens belonged to five other pathogens (*Haemophilus influenzae, Legionella pneumophila*, *Chlamydia pneumoniae*, *Mycoplasma pneumoniae* and *Streptococcus pneumoniae*) was validated by gene sequencing.

### Nucleotide sequencing

Automated sequencing of the nine genes belonging to the studied pathogens was carried out at Sangon Biotech (Shanghai, China) using an Applied Biosystems 3730xl DNA Analyzer (Thermo Fisher Scientific, Foster City, USA), following the contractor’s procedure. Briefly, preliminary amplification of the nucleic acids from the nine pathogens was completed in our laboratory by conventional RT-PCR and the products were sent for DNA sequencing. The sequences of PCR primers for sequencing are listed in Table 8. The sequencing results were compared with the sequences in GenBank using the BLAST algorithm.

**Table 8:** Sequences of PCR primers for DNA sequencing.

Pathogen	Primer	Sequence (5′–3′)	Amplicon sizes (bp)
Influenza A virus	CIVA-CXF01	TCTAACCGAGGTCGAAACGTA	390
	CIVA-CXR01-1	TTGTATATGAGGCCCATGCA	
Influenza B virus	C-IVB-CXF1-1	TTGCCTACCTGCTTTCATTGA	235
	C-IVB-CXR1	ATTCCCGATAAGGGCTCTGT	
RSV	CRSV-CXF01	TGGGGCAAATACAAAGATGGC	243
	CRSV-CXR01-1	CTTCCTAATCTRGACATAGCATAT	
Adenovirus	CADV-CXF005	ACTTGCARGACAGAAACACAGA	530
	CADV-CXR004	GAAGGGGTTGACRTTGTCCA	
	CADV-CXR004-3	GAAGGGGTTNACGTTGTCCA	
	CADV-55CXF01	AGGTGTAAAAAATGGTGAGGAG	830
	CADV-55CXR03	ATGCCATTCAGTGGAAAACAATAG	
*Legionella pneumoniae*	CLP-CXF04-1	GCTGTTATGGGGCTTGCAAT	348
	CLP-XR04	CAATACAACAACGCCTGGCTT	
*Haemophilus influenzae*	CHI-CXF003	CAAGTAAAATTTCCAGCTTGGTC	275
	CHI-CXR004	CGCATCTAAGATTTGAACGTAT	
*Mycoplasma pneumoniae*	CMP-CXF002	CGCCTCGATCCTGATTCTGT	269
	CMP-CXR002	TATCCACATCAAACCCGGTCT	
*Chlamydia pneumoniae*	CCP-CXF01	CAACAGCTACTGGAACAAAGTCT	267
	CCP-CXR02	AGGAAACAATTTGCATGAAGTCTGA	
*Streptococcus pneumoniae*	CSP-CXF01	TGAGAACGGCTTGACGATTGA	298
	CSP-CXR01	AGTGTCCTTGTACTTGACCCA	

### Statistical analysis

Consistency between the results of immunoassays, sequencing and real-time PCR assays was verified by applying Cohen’s kappa test using SPSS software, version 21.0 (IBM Corp., Armonk, NY, USA). The kappa coefficient (95% CI) value was graded as follows: 0–0.20, small; 0.21–0.40, fair; 0.41–0.60, moderate; 0.61–0.80, substantial and 0.81–1, near perfect agreement. A p value of < 0.05 was considered statistically significant.

## Supplementary Information

Supplementary Information 1.

Supplementary Information 2.

## Data Availability

Data archiving is not mandatory but will be made available from the corresponding author upon reasonable request.
