# Supplementary material for: Development of a diagnostic assay by three-tube multiplex real-time PCR for simultaneous detection of nine microorganisms causing acute respiratory infections
Source: Sci Rep. 2022 Aug 3;12:13306. doi: 10.1038/s41598-022-15543-6 (PMC9427838; doi:10.1038/s41598-022-15543-6)
Supplement: Supplementary file 1 — Supplementary Information 1. [file 41598_2022_15543_MOESM1_ESM.pdf]

Appendix: Electropherogram Data of DNA Sequencing for the detected Pathogens

DNA sequencing of nucleic acids extracted from 179 clinical specimens was performed based on Sanger's method. Sanger Sequencing was performed by Sangon Biotech and the raw data output as fluorescent peak trace chromatograms was supplied by this contractor as well. BLAST search was carried out to verify sequences producing significant alignments. Electropherogram Data was exported by using Applied Biosystems Sequence Scanner software version 1 and representative results for the corresponding pathogen identification are displayed on the following pages.

DNA Sequencing targeting Matrix Protein 2 (M2) and Matrix Protein 1 (M1) genes of Influenza A Virus Page 1-2

DNA Sequencing targeting Matrix Protein 1 (M1) genes of Influenza B Virus Page 3

DNA Sequencing targeting Human Respiratory Syncytial Virus subgroup B complete genome of Respiratory Syncytial Virus Page 4

DNA Sequencing targeting hexon gene of subtype of 1 of Adenovirus Page 5-6

DNA Sequencing targeting Human adenovirus subtype 55 complete genome of Human Adenovirus Page 7-9

DNA Sequencing targeting outer membrane P6 gene of *Haemophilus influenzae* Page 10

DNA Sequencing targeting cytadhesin protein P1 gene of *Mycoplasma pneumoniae* Page 11

DNA Sequencing targeting lyta gene of *Streptococcus pneumoniae* Page 12

Applied Biosystems

YBD2-YANG-1-CIVA-CXF01_A03.ab1

YBD2-YANG-1-CIVA-CXF01

DNA Sequencing Result for IVA Identification

KB 1.4.1.8 KB.bcp

KB_3730_POP7_BDTv3.mob

Signal: G:5787 A:7283 T:5836 C:7027 AvgSig: 6483

C#:31 W:A3 Plate Name:8110032220

TS:54 CRL:334 QV20+:331

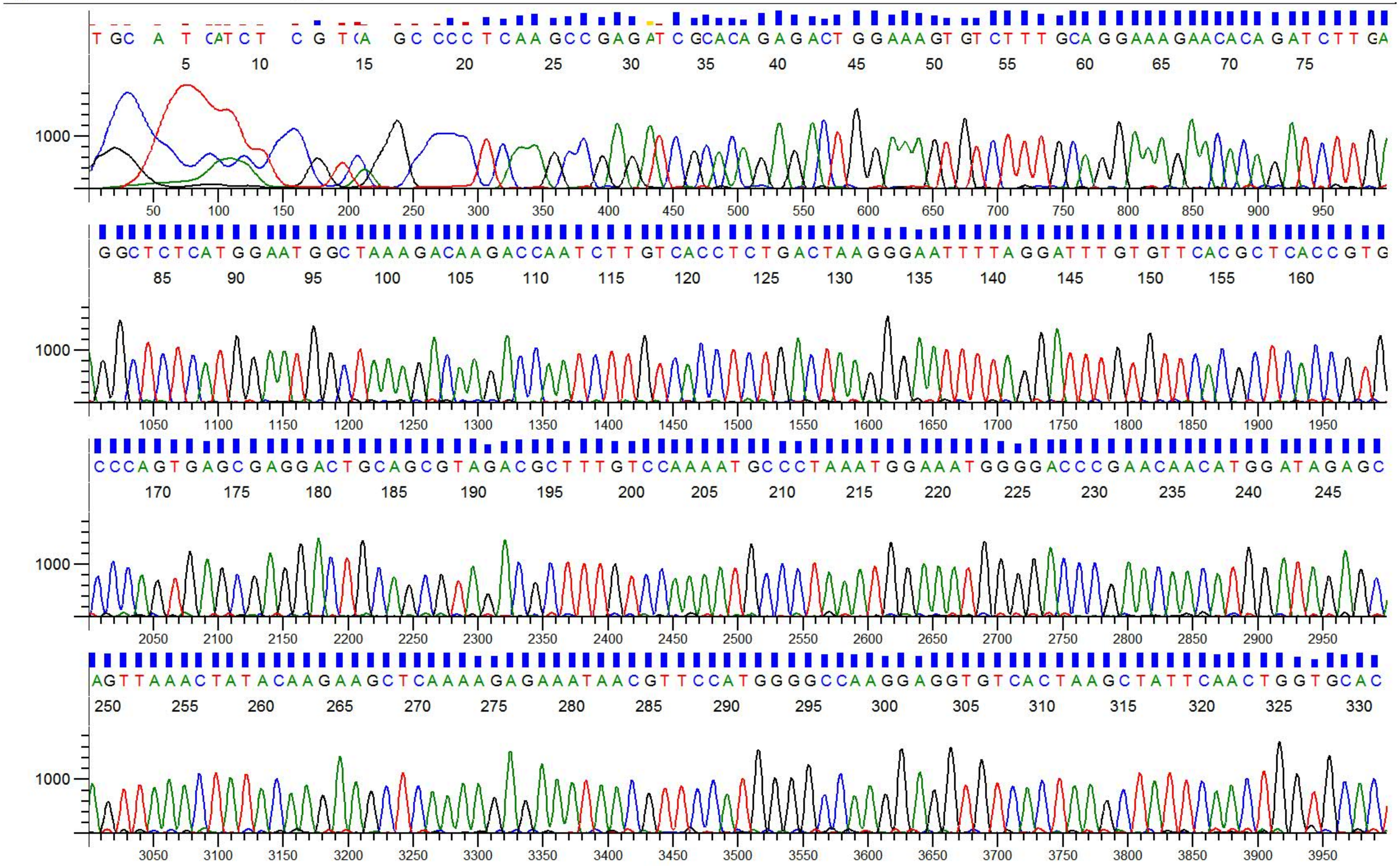

Inst Model/Name:3730xl/XE2-1414-004-1414-004

Pure Base QVs: 15 20

Mixed Base QVs: 10 16

Printed on: 十一月 26,2021 04:30:38 GMT

Sequence Scanner v1.0

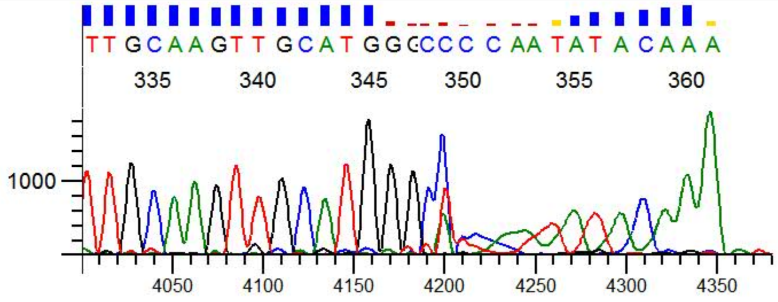
TTGCAAGTTGCATGGGCCCCAATATACAAA
335
340
345
350
355
360
1000
4050
4100
4150
4200
4250
4300
4350

Applied Biosystems

YBD2-10-2-C-IVB-CXF1-1_E03.ab1

YBD2-10-2-C-IVB-CXF1-1

DNA Sequencing Result for IVB Identification

KB 1.4.1.8 KB.bcp

KB_3730_POP7_BDTv3.mob

Signal: G:1621 A:2561 T:1876 C:1773 AvgSig: 1957

C#:23 W:E3 Plate Name:8110032220

TS:52 CRL:176 QV20+:187

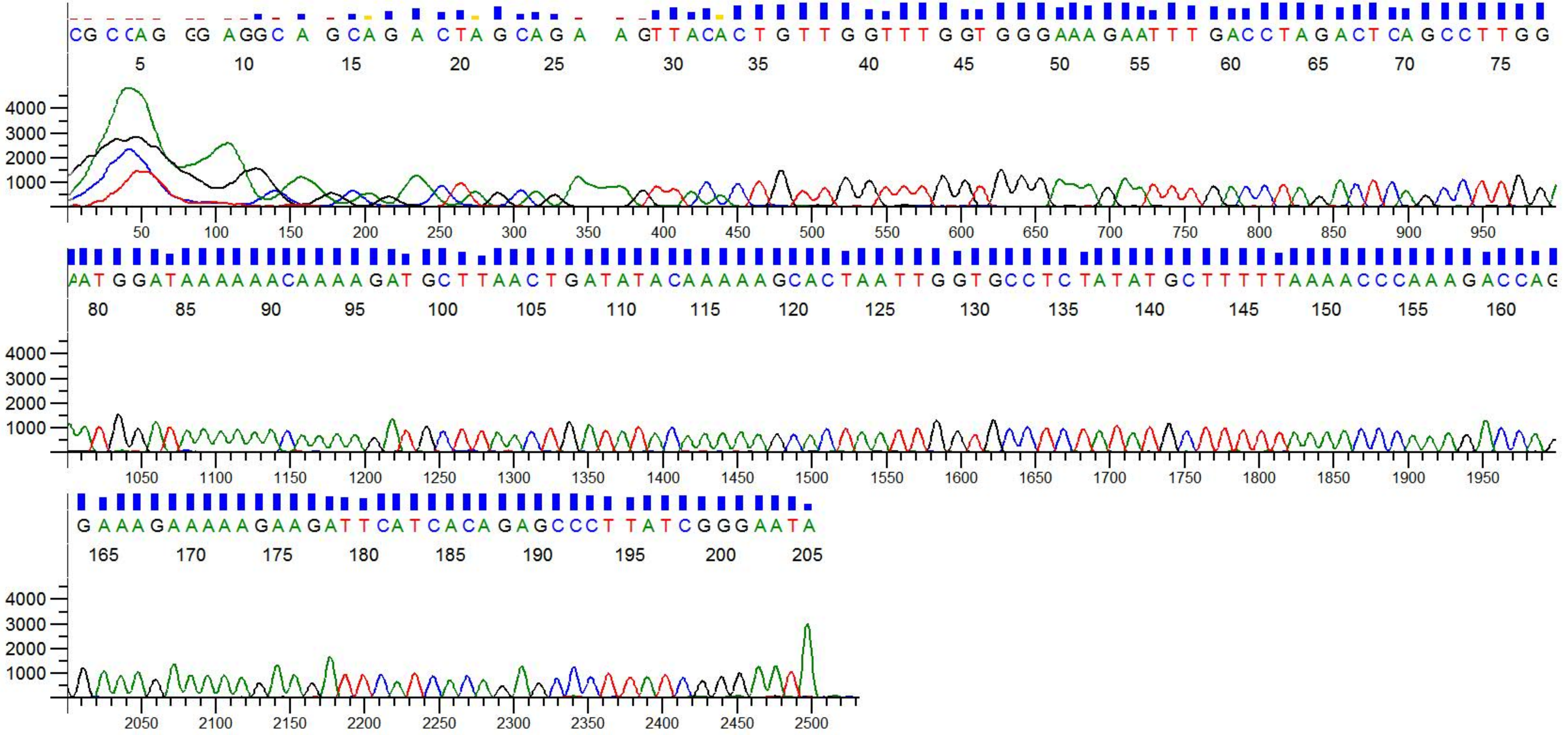

Inst Model/Name:3730xl/XE2-1414-004-1414-004

Sequence Scanner v1.0

Pure Base QVs: 15 20

Mixed Base QVs: 10 16

Printed on: 十一月 26,2021 04:30:37 GMT

Applied Biosystems

YBD2-YANG-3-CRSV-CXF01_F03.ab1

YBD2-YANG-3-CRSV-CXF01

DNA Sequencing for RSV Identification

KB 1.4.1.8 KB.bcp

KB_3730_POP7_BDTv3.mob

Signal: G:2056 A:3790 T:3816 C:2845 AvgSig: 3126

C#:21 W:F3 Plate Name:8110032220

TS:47 CRL:203 QV20+:195

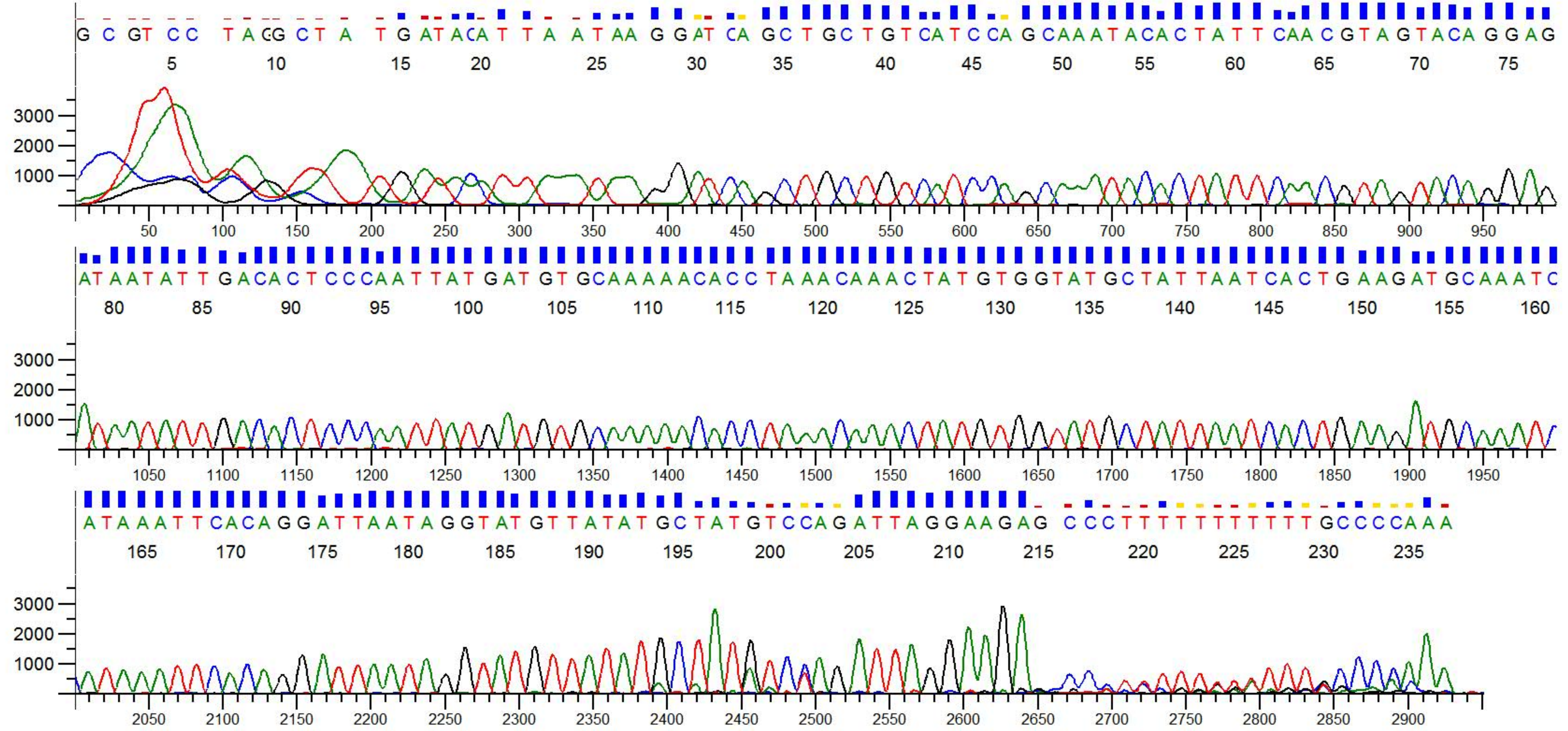

Inst Model/Name:3730xl/XE2-1414-004-1414-004

Sequence Scanner v1.0

Pure Base QVs: 15 20

Mixed Base QVs: 10 16

Printed on: 十一月 26,2021 04:30:39 GMT

Electropherogram Data Page 1 of 1

Applied Biosystems

YBD2-YANG-4-CADV-CXF005_G03.ab1

YBD2-YANG-4-CADV-CXF005

DNA Sequencing for ADV subtypes 1, 2, 3, 4, 5, 7, 46, 48 Identification

KB 1.4.1.8 KB.bcp

KB_3730_POP7_BDTv3.mob

Signal: G:2276 A:3430 T:3185 C:3062 AvgSig: 2988

C#:19 W:G3 Plate Name:8110032220

TS:53 CRL:534 QV20+:523

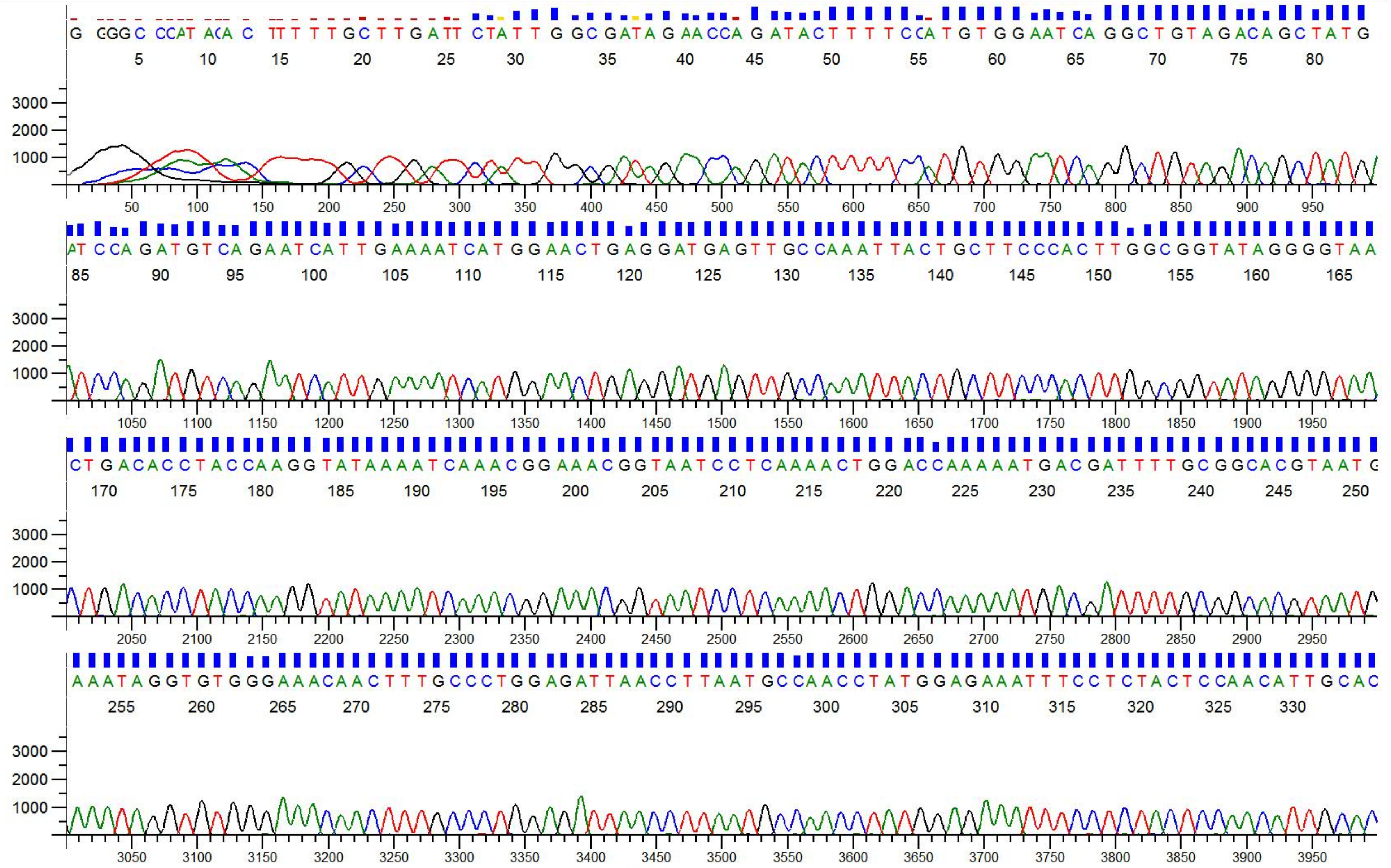

Inst Model/Name:3730xl/XE2-1414-004-1414-004

Pure Base QVs: 15 20

Mixed Base QVs: 10 16

Printed on: 十一月 26,2021 04:30:40 GMT

Sequence Scanner v1.0

Electropherogram Data Page 1 of 2

Applied Biosystems

YBD2-YANG-4-CADV-CXF005_G03.ab1

YBD2-YANG-4-CADV-CXF005

KB 1.4.1.8 KB.bcp

KB_3730_POP7_BDTv3.mob

Signal: G:2276 A:3430 T:3185 C:3062 AvgSig: 2988

C#:19 W:G3 Plate Name:8110032220

TS:53 CRL:534 QV20+:523

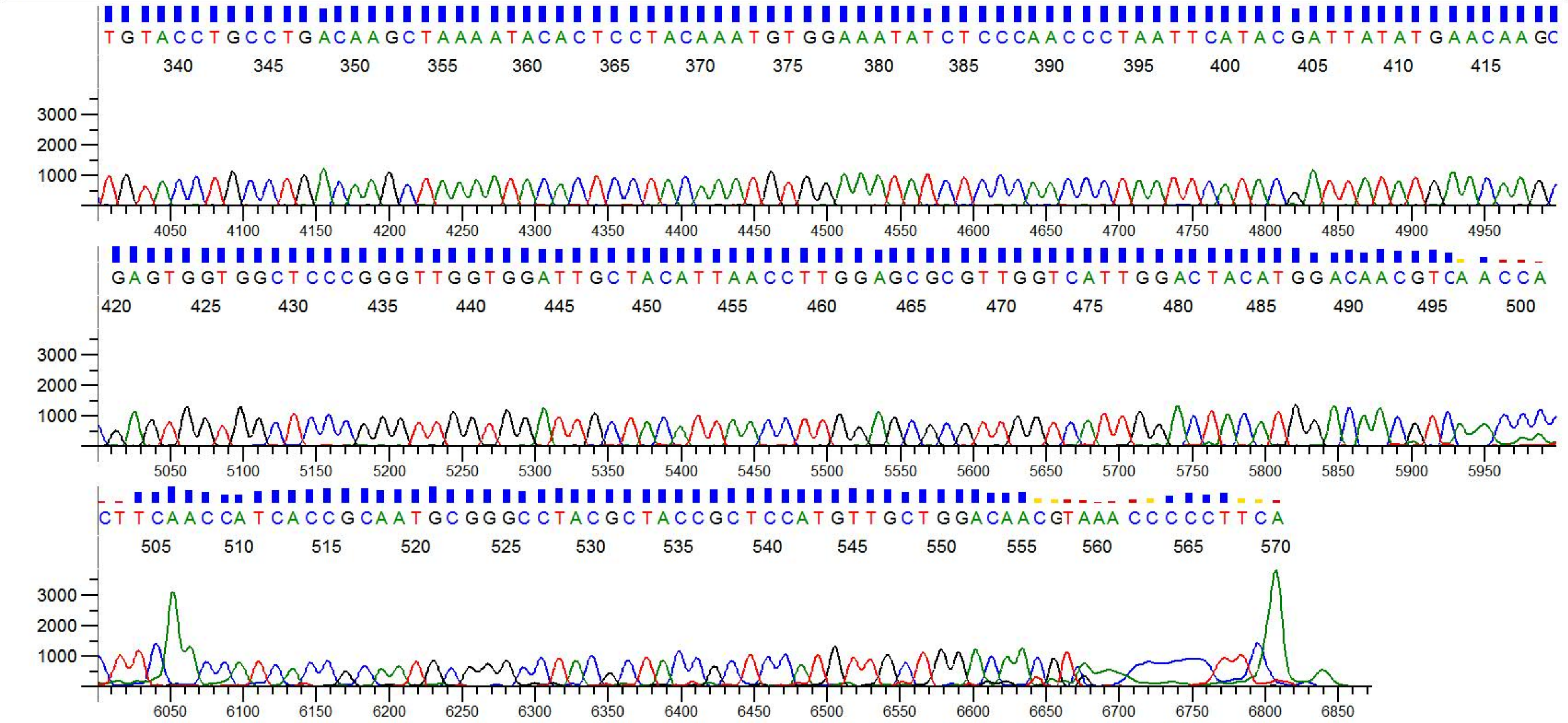

Inst Model/Name:3730xl/XE2-1414-004-1414-004

Sequence Scanner v1.0

Pure Base QVs: 15 20

Mixed Base QVs: 10 16

Printed on: 十一月 26,2021 04:30:40 GMT

DNA Sequencing for ADV subtype 55 Identification

Signal: G:2915 A:4456 T:3717 C:3739 AvgSig: 3706

C#:17 W:H3 Plate Name:8110032220

TS:49 CRL:766 QV20+:744

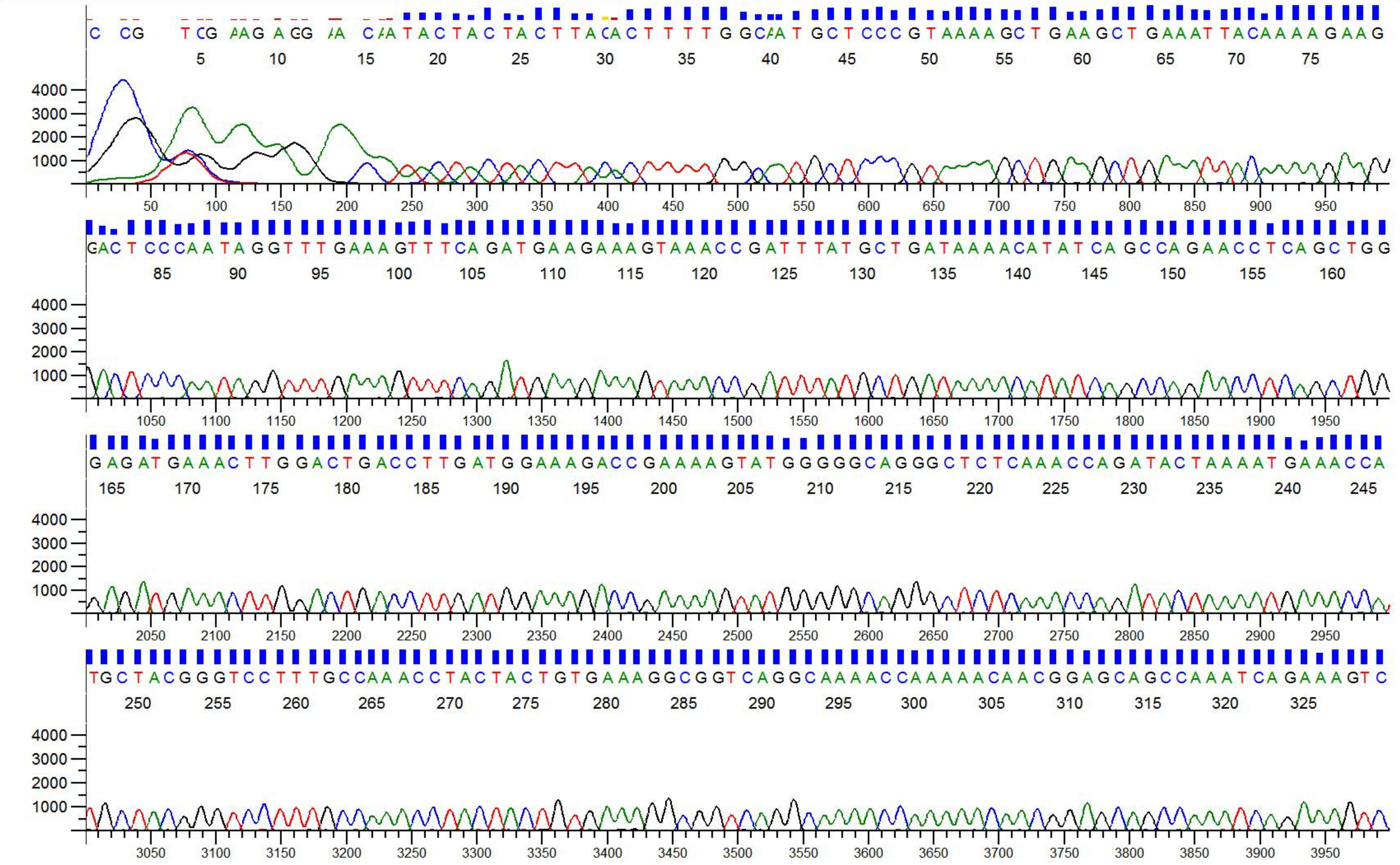

Inst Model/Name:3730xl/XE2-1414-004-1414-004

Pure Base QVs: 15 20

Mixed Base QVs: 10 16

Printed on: 十一月 26,2021 04:30:41 GMT

YBD2-YANG-5-CADV-55CXF01_H03.ab1

KB 1.4.1.8 KB.bcp

YBD2-YANG-5-CADV-55CXF01

KB_3730_POP7_BDTv3.mob

Signal: G:2915 A:4456 T:3717 C:3739 AvgSig: 3706

C#:17 W:H3 Plate Name:8110032220

TS:49 CRL:766 QV20+:744

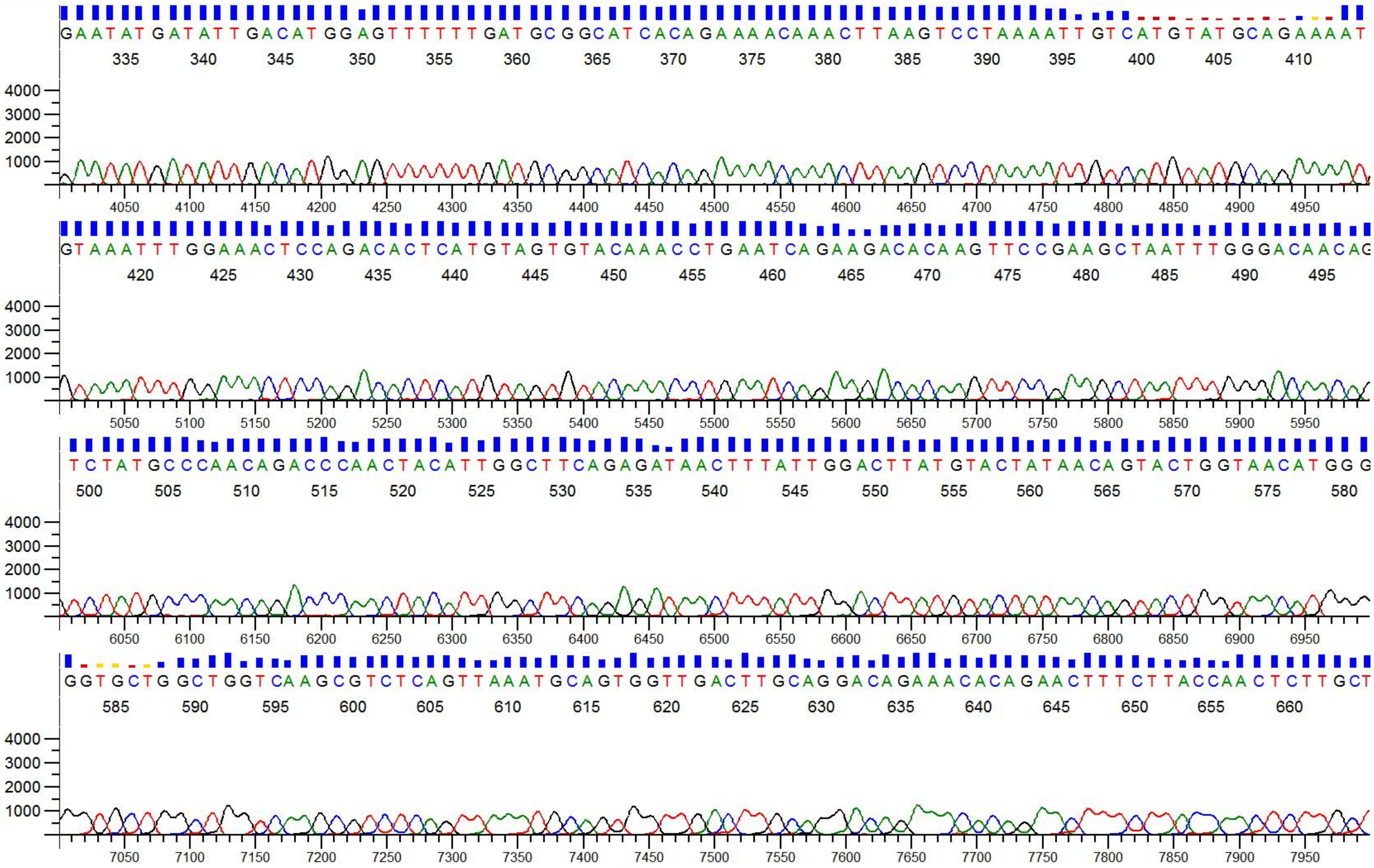

Inst Model/Name:3730xl/XE2-1414-004-1414-004

Pure Base QVs: 15 20

Mixed Base QVs: 10 16

Printed on: 十一月 26,2021 04:30:41 GMT

Sequence Scanner v1.0

Applied Biosystems

YBD2-YANG-5-CADV-55CXF01_H03.ab1

YBD2-YANG-5-CADV-55CXF01

KB 1.4.1.8 KB.bcp

KB_3730_POP7_BDTv3.mob

Signal: G:2915 A:4456 T:3717 C:3739 AvgSig: 3706

C#:17 W:H3 Plate Name:8110032220

TS:49 CRL:766 QV20+:744

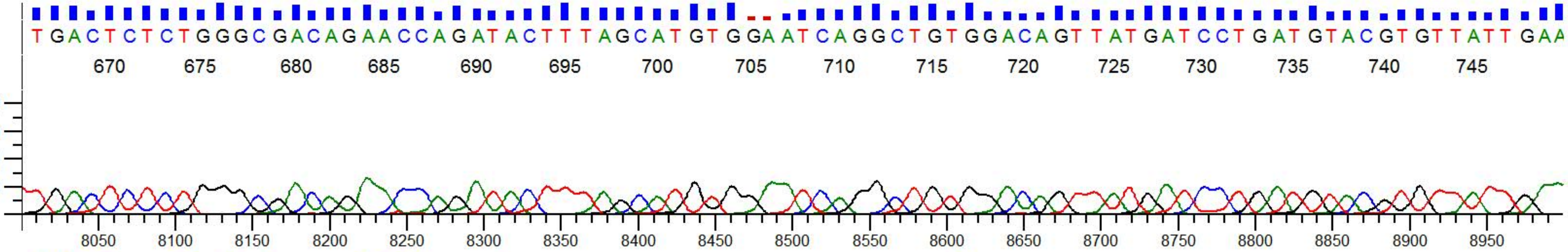

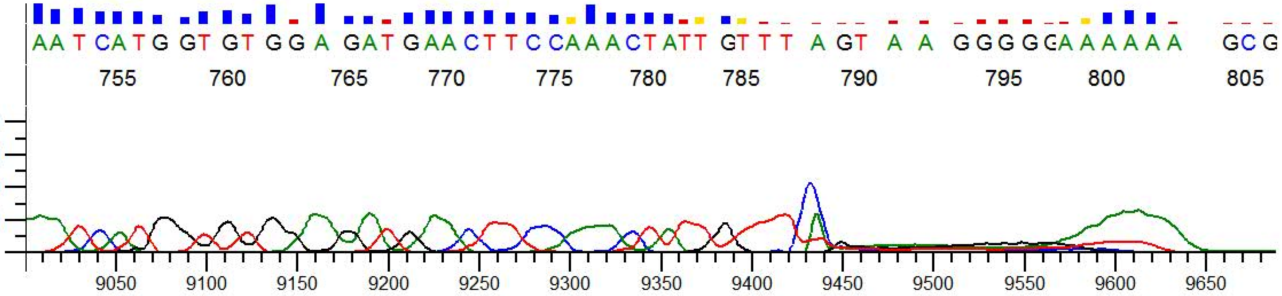

Inst Model/Name:3730xl/XE2-1414-004-1414-004

Sequence Scanner v1.0

Pure Base QVs: 15 20

Mixed Base QVs: 10 16

Printed on: 十一月 26,2021 04:30:41 GMT

Applied Biosystems

Signal: G:2849 A:2858 T:4714 C:3880 AvgSig: 3575

YBD2-YANG-7-CHI-CXF003_C04.ab1

YBD2-YANG-7-CHI-CXF003

C#:28 W:C4 Plate Name:8110032220

DNA Sequencing for *Haemophilus influenzae* Identification

KB 1.4.1.8 KB.bcp

KB_3730_POP7_BDTv3.mob

TS:54 CRL:221 QV20+:223

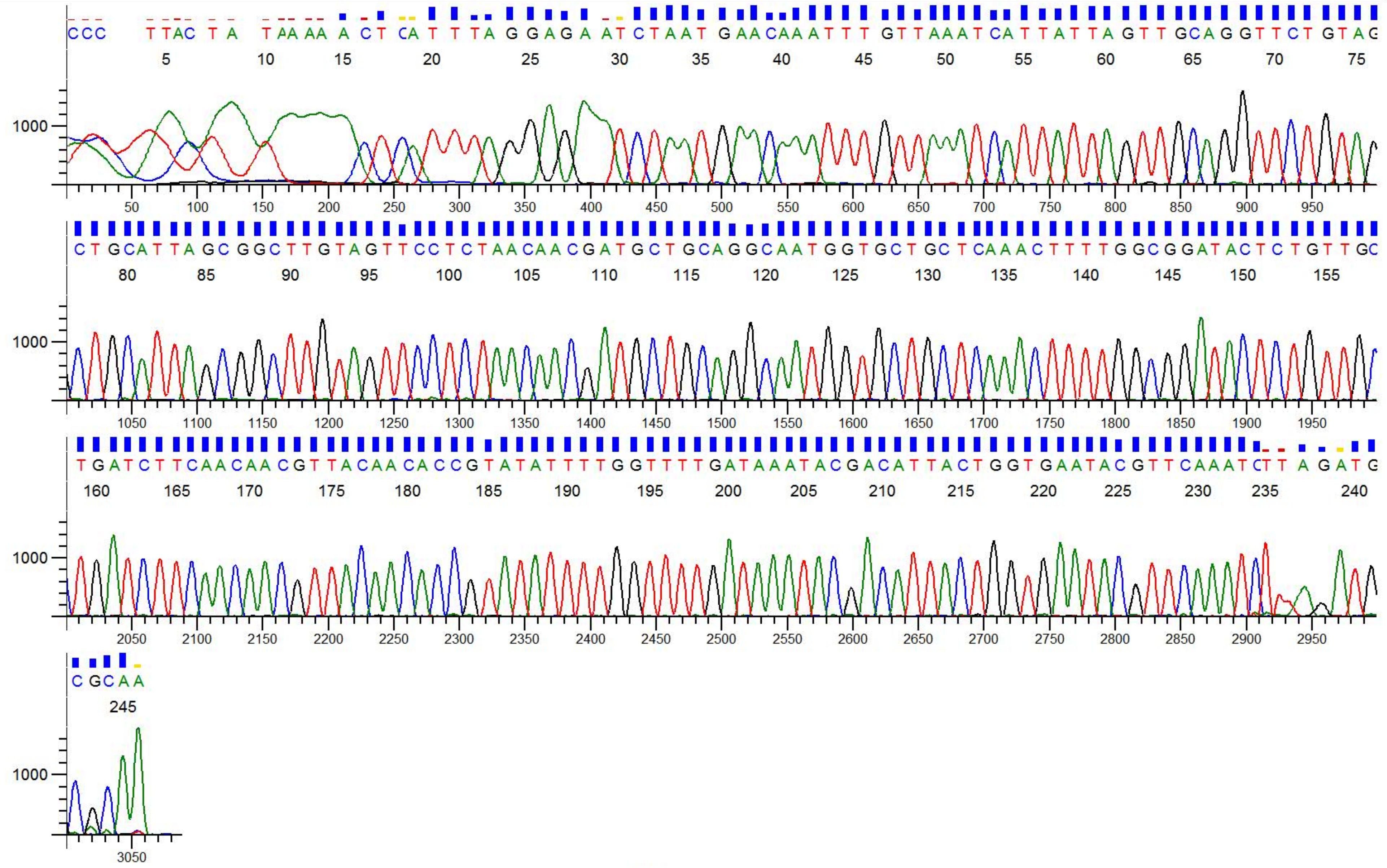

Inst Model/Name:3730xl/XE2-1414-004-1414-004

Sequence Scanner v1.0

Pure Base QVs: 15 20

Mixed Base QVs: 10 16

Printed on: 十一月 26,2021 04:30:44 GMT

Electropherogram Data Page 1 of 1

Applied Biosystems

YBD2-YANG-9-CMP-CXF002_B05.ab1

YBD2-YANG-9-CMP-CXF002

C#:45 W:B5 Plate Name:8110032220

DNA Sequencing for *Mycoplasma pneumoniae* Identification

KB 1.4.1.8 KB.bcp

KB_3730_POP7_BDTv3.mob

TS:53 CRL:214 QV20+:221

Signal: G:4751 A:4312 T:3067 C:5302 AvgSig: 4358

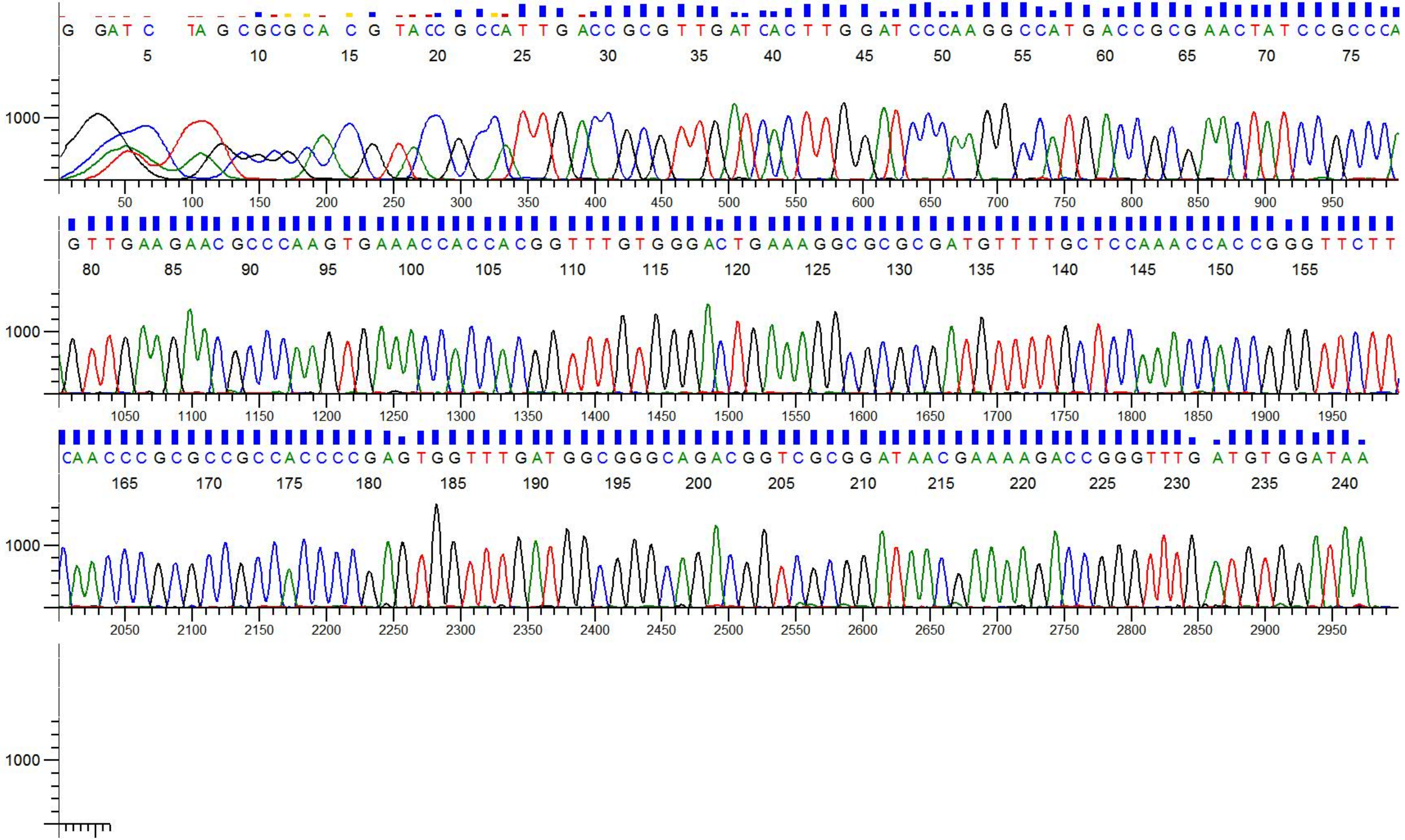

Inst Model/Name:3730xl/XE2-1414-004-1414-004

Sequence Scanner v1.0

Pure Base QVs: 15 20

Mixed Base QVs: 10 16

Printed on: 十一月 26,2021 04:30:36 GMT

Electropherogram Data Page 1 of 1

Applied Biosystems

YBD2-YANG-10-CSP-CXF01_D05.ab1 | YBD2-YANG-10-CSP-CXF01 | C#:41 W:D5 Plate Name:8110032220

DNA Sequencing for *Streptococcus pneumoniae* Identification

KB 1.4.1.8 KB.bcp
KB_3730_POP7_BDTv3.mob
TS:51 CRL:240 QV20+:240

Signal: G:4157 A:5093 T:4201 C:4537 AvgSig: 4497

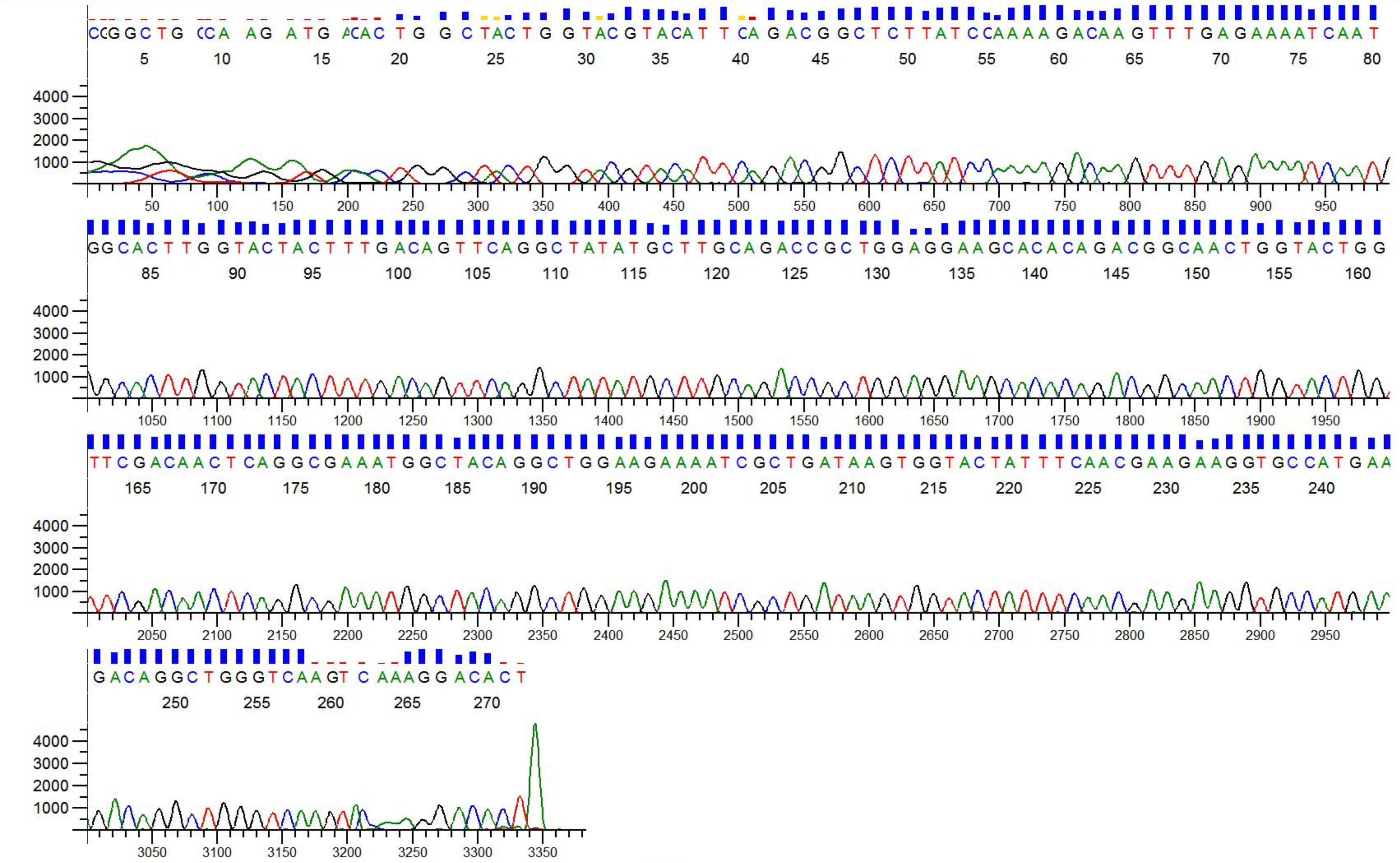

Inst Model/Name:3730xl/XE2-1414-004-1414-004

Sequence Scanner v1.0

Pure Base QVs: 15 20

Mixed Base QVs: 10 16

Printed on: 十一月 26,2021 04:30:37 GMT

Electropherogram Data Page 1 of 1
