# Supplementary material for: Development of a diagnostic assay by three-tube multiplex real-time PCR for simultaneous detection of nine microorganisms causing acute respiratory infections
Source: Sci Rep. 2022 Aug 3;12:13306. doi: 10.1038/s41598-022-15543-6 (PMC9427838; doi:10.1038/s41598-022-15543-6)
Supplement: Supplementary file 2 — Supplementary Information 2. [file 41598_2022_15543_MOESM2_ESM.docx]

Figure S1


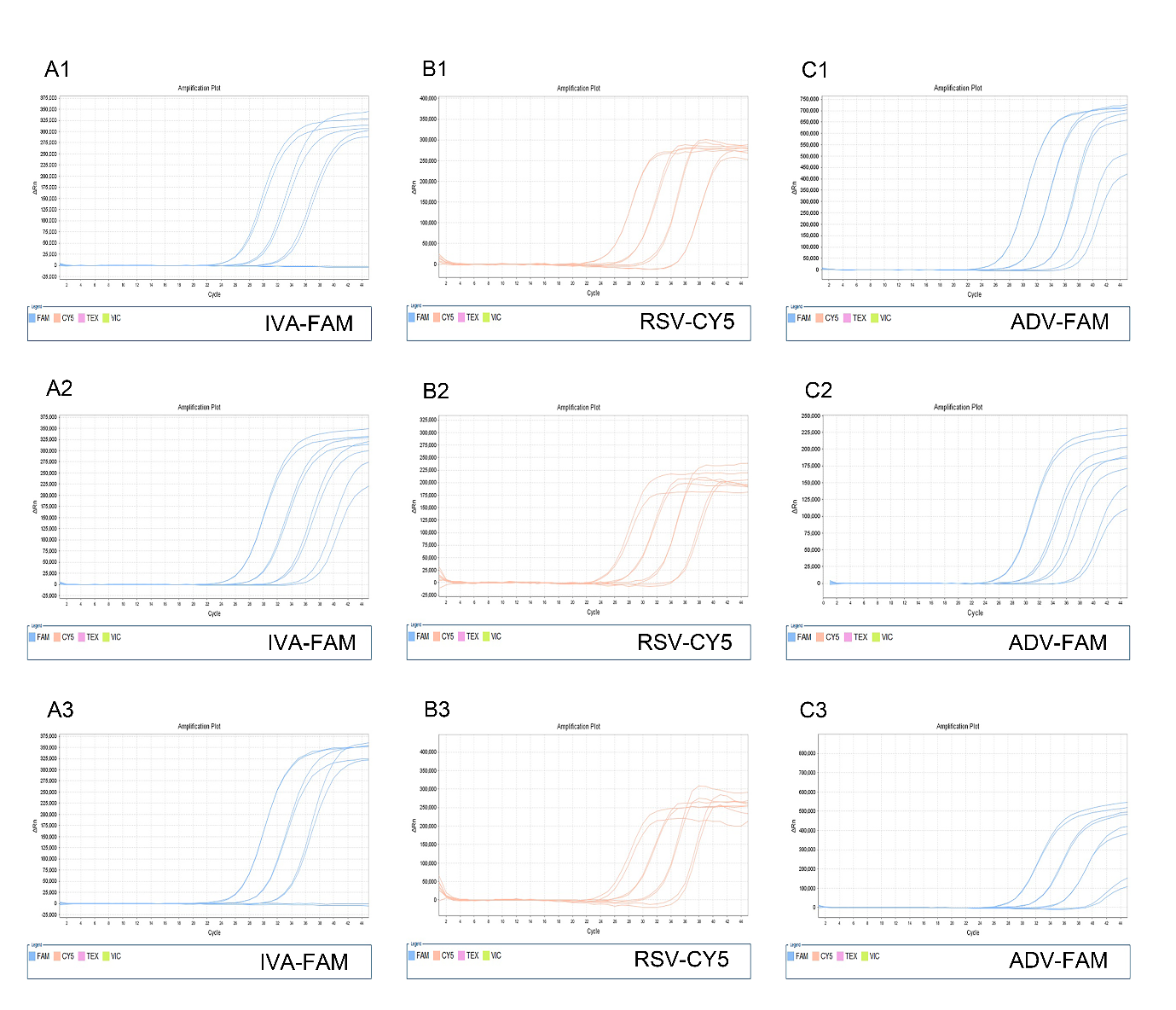


**Figure S1.** Optimised reverse transcriptase concentrations and primer annealing temperatures in the multiplex real-time PCR. A1, A2 and A3 are the amplification curves of IVA under varied reverse transcriptase concentrations (0.24 U/µl, 0.4 U/µl and 0.8 U/µl, respectively. The data from this showed a concentration of 0.4 U/µl MMLV reverse transcriptase generated a full range of detection for RNA pathogens). B1, C1, B2, C2, B3 and C3 are the amplification curves of RSV and ADV, respectively, under annealing temperatures of 55ºC, 58 ºC and 60 ºC. The Tm analysis of amplicons revealed that 55ºC generated the highest fluorescence intensity of amplification plots and produced curves with standard shapes.

Figure S2


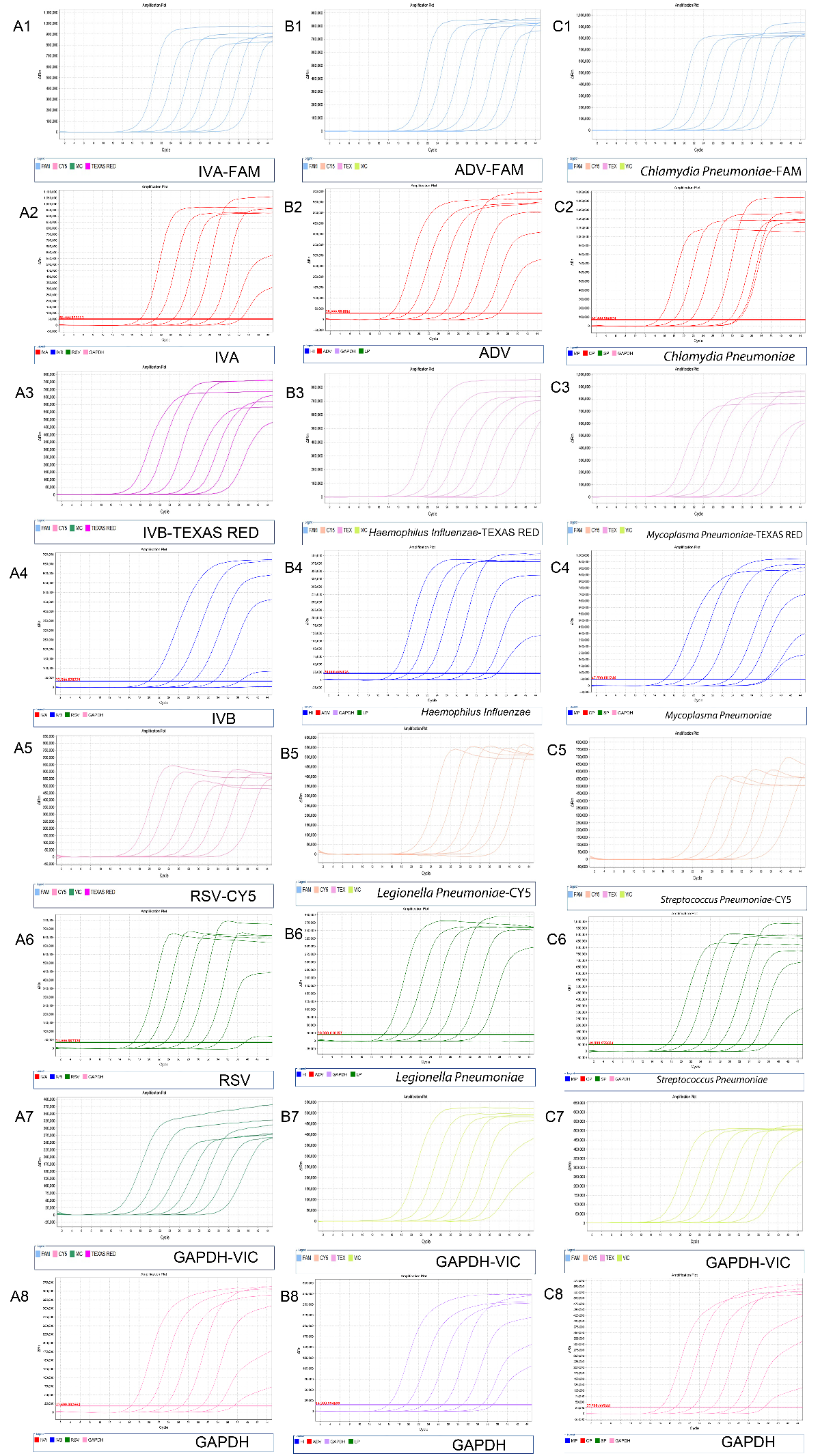


**Figure S2.** Amplification curve profiles of multiplex real-time PCR. A1-A6, B1-B6 and C1-C6 show that FAM, TEXAS RED and Cy5 fluorescent signals were generated only by the target pathogens and A7-A8, B7-B8 and C7-C8 show VIC fluorescent signals were generated by the DNA of the internal reference gene GAPDH for all existing pathogens by the three-tube real-time RT-PCR system. A-C, 1, 3, 5 and 7, and A-C, 2, 4, 6 and 8 show the amplification curves obtained under the conditions of template nucleic acids from nine individual pathogens and a mixture of all pathogens, respectively.
